# Giardia antagonizes beneficial functions of indigenous and therapeutic intestinal bacteria during protein deficiency

**DOI:** 10.1080/19490976.2024.2421623

**Published:** 2024-11-05

**Authors:** Aadra P. Bhatt, Jason W. Arnold, Muyiwa Awoniyi, Shan Sun, Verônica Feijoli Santiago, Deniz Coskuner, Pedro Henrique Quintela, Kenneth Walsh, Jamie Xiao, Renay Ngobeni-Nyambi, Brenna Hansen, Ajay S. Gulati, Ian M. Carroll, M. Andrea Azcarate-Peril, Anthony A. Fodor, Jonathan Swann, Luther A. Bartelt

**Affiliations:** a Division of Gastroenterology and Hepatology, Department of Medicine, and Lineberger Comprehensive Cancer Center, University of North Carolina at Chapel Hill, Chapel Hill, NC, USA; b Center for Gastrointestinal Biology and Disease, University of North Carolina at Chapel Hill, Chapel Hill, NC, USA; c Department of Molecular Genetics and Microbiology, Duke Microbiome Center, Duke University School of Medicine, Durham, North Carolina, USA; d Department of Gastroenterology Hepatology and Nutrition, Digestive Diseases and Surgery Institute of the Cleveland Clinic Foundation, and Department of Inflammation and Immunity, Lerner Research Institute, Cleveland Clinic, Cleveland, Ohio, USA; e Department of Bioinformatics and Genomics, University of North Carolina at Charlotte, Charlotte, NC, USA; f School of Human Development and Health, Faculty of Medicine, University of Southampton, Southampton, UK; g Institute of Biomedicine, Federal University of Ceará, Fortaleza, CE, Brazil; h Division of Infectious Diseases, Department of Medicine, University of North Carolina at Chapel Hill, Chapel Hill, NC, USA; i Department of Microbiology, Stellenbosch University, Stellenbosch, South Africa; j Department of Pediatrics, University of North Carolina at Chapel Hill, Chapel Hill, NC, USA; k Department of Nutrition, Gillings School of Public Health, University of North Carolina at Chapel Hill, Chapel Hill, NC, USA; l Department of Pathology and Laboratory Medicine, University of North Carolina at Chapel Hill, Chapel Hill, NC, USA; m Microbiome Core, University of North Carolina at Chapel Hill, Chapel Hill, NC, USA; n Department of Microbiology & Immunology, University of North Carolina at Chapel Hill, Chapel Hill, NC, USA

**Keywords:** Giardia, probiotics, malnourishment, bile acids, gnotobiotic models

## Abstract

Undernutrition in children commonly disrupts the structure and function of the small intestinal microbial community, leading to enteropathies, compromised metabolic health, and impaired growth and development. The mechanisms by which diet and microbes mediate the balance between commensal and pathogenic intestinal flora remain elusive. In a murine model of undernutrition, we investigated the direct interactions *Giardia lamblia*, a prevalent small intestinal pathogen, on indigenous microbiota and specifically on Lactobacillus strains known for their mucosal and growth homeostatic properties. Our research reveals that *Giardia* colonization shifts the balance of lactic acid bacteria, causing a relative decrease in *Lactobacillus spp*. and an increase in *Bifidobacterium spp*. This alteration corresponds with a decrease in multiple indicators of mucosal and nutritional homeostasis. Additionally, protein-deficient conditions coupled with *Giardia* infection exacerbate the rise of primary bile acids and susceptibility to bile acid-induced intestinal barrier damage. In epithelial cell monolayers, *Lactobacillus spp*. mitigated bile acid-induced permeability, showing strain-dependent protective effects. *In vivo, L. plantarum*, either alone or within a *Lactobacillus* spp consortium, facilitated growth in protein-deficient mice, an effect attenuated by *Giardia*, despite not inhibiting Lactobacillus colonization. These results highlight Giardia’s potential role as a disruptor of probiotic functional activity, underscoring the imperative for further research into the complex interactions between parasites and bacteria under conditions of nutritional deficiency.

## Introduction

Childhood undernutrition and linear growth impairment are a widespread and complex global health problem^1,2^. Beyond a state of nutritional deficiency, our current understanding suggests that childhood undernutrition also includes nutrient-dependent disruption in the absorptive and metabolic functions of the developing small intestine (SI).^3^ These functions are known to be influenced by the density, composition, and function of resident intestinal microbes. Although nutrient absorption predominantly occurs in the SI, most studies examining the influences of intestinal microbiota on host nutritional and metabolic homeostasis utilize platform technologies that primarily profile colonic microbial communities. Recent microbial community profiling of duodenal aspirates and biopsies collected from undernourished children unresponsive to nutritional supplementation revealed a shift toward predominantly oral-mucosal bacteria^4,5^ and notably a reduction in prototypic SI commensals like *Lactobacillus spp*.^6^ Additionally, conventional intestinal pathogens, such as diarrheagenic *Escherichia coli* types, *Campylobacter spp*. and *Giardia lamblia* that independently associate with impaired childhood growth^7^ have also been detected in SI communities from treatment-refractory undernourished children.^5,8^ The potential consequences of interactions between the co-occurrence of these conventional gut pathogens and altered SI microbial communities in children with undernutrition is poorly understood.

*G. lamblia* (*Giardia*) is implicated in several studies examining the consequences of disrupted SI microbial communities on childhood growth and intestinal function. We and others have shown that *Giardia* may restrict child linear growth through dose-dependent SI epithelial cell permeability dysfunction and disruptions in microbial-host nutrient homeostasis.^9,10^ *Giardia* also associates with markers of SI bacterial overgrowth^11^ and diminished markers of lymphocyte activation.^8^ Using gnotobiotic wild-type mice, we have also shown that *Giardia*-mediated growth impairment results from the convergence of two independent factors: inadequate protein intake, and perturbed intestinal microbiota.^9,10^ These data link microbiota-host homeostasis with the presence of *Giardia*. However, the directionality of this interaction, which specific bacteria are most relevant, and the consequences of these disruptions on intestinal development and overall growth trajectories are not well understood.

Trials to remediate disrupted intestinal microbiota and restore healthy child growth are underway (e.g. NCT05570045, NCT00118872). Among the different strategies, probiotic interventions with defined bacteria, like certain *Lactobacillus spp*. strains have been appealing. In animal models, specific *Lactobacillus spp*. probiotics have been shown to support linear growth in mono-associated undernourished mice,^12^ protect intestinal epithelial cell integrity and support epithelial repair,^13,14^ and regulate mucosal and immune responses to pathogens like *Giardia*.^15–17^ However, undernutrition can lead to rapid metabolic adaptations in *Lactobacillus spp*. and potential diminished mucosal-associated compartmentalization^18^ that could limit beneficial host interactions from commensal and/or probiotic strains.

Here, we investigated interactions between *Giardia* and specific *Lactobacillus spp*. in the nourished and undernourished murine host. We find that during protein deprivation, *Giardia* contributes to specific reductions in specific *Lactobacillus* species abundance, relative to other commensal bacteria. These changes are coupled with decreases in indicators of disrupted intestinal mucosal and nutrient homeostasis. We find that protein undernutrition increases intestinal primary bile acids, and the additional presence of *Giardia* associates with indicators of increased bile acid-associated intestinal epithelial cell (IEC) injury. In an *in vitro* model of IEC monolayers, bile acids cause permeability defects at physiological concentrations, but a bile salt hydrolase-expressing *L. plantarum* WCSF1 protects against this injury. While a consortium of commensal *Lactobacillus spp*. or mono-association with *L. plantarum* WCSF1 supports weight gain in gnotobiotic protein deprived mice, co-colonization with *Giardia* inhibits this effect. These findings indicate that *Giardia* colonization may contribute to loss of microbiota-host homeostasis through loss of commensal *Lactobacillus* functions. These findings implicate an important role for *Giardia* modulation of small intestinal microbiota composition, particularly in the context of engraftment and/or efficacy of bacterial-based therapies and live biotherapeutic products (LBPs) used to correct malnutrition.

## Materials and methods

### Mice

Mouse experiments were conducted in strict accordance with recommendations in the Guide for the Care and Use of Laboratory Animals of the National Institutes of Health. The mouse experimental protocol was approved by the Institutional Animal Care and Use Committee at the University of North Carolina at Chapel Hill (IACUC Protocol# 23–126).

For specific pathogen free (SPF) mouse experiments, all experiments used male wild-type C57Bl/6 J mice obtained from Jackson Laboratories. These mice were age-matched at 21 days of life and at least 10 g prior to shipping. Mice were housed in light cycles of 12:12 (12 h light and 12 h dark), at the set temperature of 72 °F (±3 °F) and a set humidity range of 30–70%. Mice were housed 1–2 mice/cage in individually ventilated cages (IVCs) in the Division of Comparative Medicine BSL2 isolation cubicle facility at UNC-CH. For experiments in germ-free (GF) mice, all experiments used both male and female C57Bl/6 J wild-type and *Rag2*^*−/−*^ (C57Bl/6 J background) mice at 8–16 weeks-old obtained from the National Gnotobiotic Rodent Resource Center at UNC-CH. For each experiment, mice were sorted into age and sex-matched intervention and control groups and singly housed upon transfer from isolators to the SPF cubicle facility. Mouse weights were obtained using a battery-operated digital scale (Ohaus) with a precision of ±0.01 g. Fecal pellets were obtained as previously described.^9,10,19^

### Mouse diets

Mice were fed either the Protein deficient (PD) diet (Envigo, TD.110200) or the isocaloric control (CD) diets (Envigo, TD.08678). For experiments in SPF mice, mice were acclimated immediately onto their respective diets upon arrival. For experiments in wild-type GF mice, mice were acclimated to the PD diet while in the isolator environment for 1–2 weeks prior to microbial challenges in the Division of Comparative Medicine BSL2 isolation cubicle facility at UNC-CH. For experiments in *Rag2*^*−/−*^ GF mice, mice were transferred to the cubicle facility and given microbial challenge prior to transition to the PD diet five days later as indicated in Figure 6.

### Giardia lamblia cyst preparation and colonization

For experiments in SPF mice, ultra-purified *G. lamblia* (assemblage B, H3 cysts) were acquired from Waterborne, Inc and as previously described.^9^ Cysts were used within 48 days of arrival and rinsed in PBS 3 times using centrifugation at 400–600 g ×10 minutes. Cysts were counted by hemacytometer and diluted in PBS as necessary for an inoculum of 10^6^ cysts/mL. Mice were challenged with 100 μl by oral gavage for a challenge dose of 10^5^ cysts/mouse. For mono-association or direct co-colonization with *Lactobacillus* experiments, axenic *G. lamblia* (assemblage B, H3 cysts) were acquired from *Giardia* mono-associated *Rag2*^*-/-*^ (C57Bl/6) propagators in the National Gnotobiotic Rodent Resource Center at UNC-CH. Cysts were prepared from fresh fecal homogenates using 400 μl PBS per fecal sample, followed by gravity sedimentation and dilution to 10^4^ cysts/100 μl. Mice were challenged with 100 μl each.

### Lactobacillus strains, in vitro growth curves, and in vitro permeability assays

The strains listed in Table 1 were acquired as indicated and stored in glycerol stocks according to manufacturer protocols: *L. plantarum* WCSF-1 (BAA-793), *L.johnsonii* (332), *L. casei* (334). *L. rhamnosus*_AMC143 and *L.rhamnosus*_AMC010 were obtained from glycerol-preserved archived strains in the UNC Microbiome Core after isolation as previously described.^21^ De Man – Rogosa – Sharpe (MRS) media was obtained from Gibco and mixed as either broth or agar according to manufacturer instructions. TYI-S-33 media that contains 1% w/v bile mix (Sigma Aldrich S8381), but modified to be antibiotic-free was made as previously described.^19^ Individual colonies isolated on MRS agar plates were used to inoculate liquid MRS or TYI-S-33 media. 200 μl of inoculated culture was placed into 96 well plates in triplicate. Automated optical density (OD) readings were measured every 15 min for 22.5 h using a closed-system, stationary TECAN plate reader set at 37°C in ambient atmosphere.

**Table 1.** List of strains and sources.

Strain	Source
*L. plantarum* WCSF-1 (BAA-793)^20^	ATCC
*L. johnsonii* (332)	ATCC
*L. casei* (334)	ATCC
*L. rhamnosus*_AMC143	UNC Microbiome Core^21^
*L.rhamnosus*_AMC010	UNC Microbiome Core^21^

For *in vitro* assessment of epithelial cell integrity, sterilized conditioned media were used: *L. rhamnosus*_AMC143 and *L. plantarum* WCSF-1 were cultured in antibiotic-free TYI-S-33 media until OD600 reached 0.6–0.8. Cultures were centrifuged for 15 min at 6000 × g in a refrigerated Sorvall RC-5C high speed centrifuge. The pH of supernatant was verified to be within 7.0–7.2, then sterilized by sequential filtration through 0.45 and 0.2 micron PES syringe filters (VWR).

### Bile tolerance assessment of Lactobacillus strains

Strains listed in Table 1 were cultured in MRS agar containing 0.2% w/v final concentration of TCA, GCA, TDCA or GDCA (Sigma Aldrich) using a previously reported method.^22^ Briefly, autoclaved 2× MRS agar was uniformly mixed with equal volume of sterilized solution of bile acid (0.4% w/v) prepared in deionized water and poured into petri plates. Once solidified, these were transferred to an anaerobic chamber (Bactron, Sheldon Manufacturing) for 24 h at room temperature, and then indicated *Lactobacilli* were streaked using sterile disposable loops; bacteria were cultured anaerobically at 37°C for 5 days after which growth was visually assessed in a semiquantitative manner; growth in standard MRS agar was the benchmark for normalizing growth in media supplemented with the indicated bile acid being tested. Once growth enumeration was complete, bacterial colonies were scraped off using sterile plastic L-shaped cell spreader (VWR Scientific) and surface of agar was rinsed by squirting sterile distilled water using a polyethylene wash bottle. Once plates were confirmed to be free of visible bacterial colonies using a dissection microscope (Olympus), plates containing deconjugated bile acids evident as cloudy halos in media were photographed using an iBright imager (Invitrogen).

### Lactobacillus growth for preparation for mouse colonization

Single colonies of each *Lactobacillus* strain were selected from MRS agar plates and grown overnight in MRS broth at 37°C under ambient atmosphere. Overnight cultures were back-diluted (1:100 dilution) in 10 ml MRS broth and incubated to early log phase growth (OD 0.2–0.4) at 37°C under ambient atmosphere. Bacterial cells were harvested by centrifugation, washed with PBS, and resuspended in PBS at a concentration of 10^7^ cells/ml. Mice were then challenged with 100 μl each per oral gastric gavage for a challenge inoculum of 10^6^ bacterial cells per strain per mouse.

### Fecal microbiota transplant

To transfer fecal microbiota from SPF PD-fed uninfected mice, fresh fecal pellets were collected serially between 7 and 21 days on diet. The pellets were then snap-frozen and stored at − 80°C. On the day of challenge, frozen fecal pellets were pooled, thawed, diluted in PBS (10 mg/mL of PBS), and homogenized. Mice were inoculated with 100 *μ*l of the homogenate by orogastric gavage as previously described.^9^

### 16S bacterial profiling

DNA was extracted from stool as previously described.^23^ Fecal 16S rRNA amplicon libraries were prepared using Illumina Nextera two-stage PCR library protocol. Briefly, the 515F-806 R primer set containing Illumina adaptors were used to run a limited cycle PCR (25 cycles), after which the amplicons were cleaned up using Axygen Ampure PCR Cleanup Beads. Cleaned amplicons were then subject to indexing using Nextera barcodes in an 8-cycle PCR. Barcoded Libraries were cleaned up using Axygen Ampure PCR cleanup beads and quantified using PicoGreen DS DNA reagent. Libraries were pooled at equimolar concentration and subject to sequencing on Illumina MiSeq 2 × 250platform. Sequencing reads were analyzed using DADA2^24^ and QIIME2.^25^ The forward reads were truncated to 220bp and denoised with DADA2. Chimera were removed using the pooled method. The amplicon sequence variants (ASV) were classified based on the SILVA database (release 138.1). The ASV abundance table was normalized as previously described^26^ to correct for different sequencing depth across samples.

### Detection and quantification of intestinal microbes and host gene expression

#### Giardia:

*Giardia* trophozoites were detected by light microscopy and enumerated with a hemocytometer as previously described.^9^ Briefly, a small intestinal fragment of 4 cm in length was taken 1 cm from the pyloric sphincter (duodenum) and then opened longitudinally, minced, and placed in 4 mL of ice-cold PBS for 30–45 min. Trophozoites were counted in 10 μl aliquots using a hemocytometer.

#### Lactobacillus:

For culture detection and enumeration of *Lactobacillus* from gnotobiotic mice, the same segment that was used for *Giardia* detection was homogenized and plated using serial dilutions on MRS agar. For colonic *Lactobacillus* culture detection and enumeration, a 4 cm in length segment of distal colon was similarly obtained from each mouse. The colon contents were rinsed with ice-cold PBS and the rinsed segment was minced and homogenized prior to plating on MRS agar using serial dilutions as outlined above. Colony counts were obtained using a ProtoCOL automated colony counter after 48 h incubation in 37°C in ambient atmosphere.

#### DNA extraction from fecal and intestinal segments:

DNA extractions for all fecal samples were performed using a modified version of Qiagen MagAttract Microbial DNA Kit on KingFisher Flex instrument as previously described. DNA was extracted from duodenum samples using a modified Qiagen QIAamp DNA Extraction kit including lysis performed in ATL Lysis Buffer with Proteinase K and AL buffer. Lysate was loaded onto silica column and washed with AW1 and AW2 buffers, prior to elution and quantification as previously described.^21^

#### Lactobacillus and Bifidobacterium qPCR:

We performed targeted qPCR using previously reported primers^27^ to quantify *Lactobacillus* and *Bifidobacterium* from fecal and duodenum DNA extracts. Equivalent amounts of genomic DNA was amplified in duplicate or triplicate in 10 µL reactions consisting of 5 µL PowerSYBR master mix, 1 µL template DNA, 1 µL of Primer mix at 1 µM concentration, and PCR-grade water for fecal samples and a 1:100 dilution of template DNA from duodenum samples to dilute interfering host DNA. Amplification was performed on QuantStudio Q6 Flex qPCR instrument, and quality was confirmed by melt curve analysis. Abundance of *Lactobacillus* and *Bifidobacterium* was determined using the delta delta Ct method, with universal 16S V4 used for normalization.

#### Intestinal gene expression from mice:

For host gene expression from mice, jejunal segments (2–4 cm in length) were obtained immediately distal to the duodenum segment used for trophozoite detection. RNA extraction and cDNA synthesis were performed using QIAgen kits per the manufacturer’s directions and performed qPCR on a Quantstudio RealTime PCR machine (ThermoFisher) using SYBR Green. qPCR was performed using SYBR Green Primers for *RegIIIγ*, *MMP7*, *IL-22, Pept1, 18S, FXR*^28^, and *TGR5*^29^ are listed in Table 2, and were purchased from Sigma. Gene expression was normalized to the housekeeping gene β-actin, except for FXR and TGR5 which were normalized to 18S gene. In both instances, subsequent quantification was completed by the delta delta method using PBS control mice as a reference.

**Table 2.** List of qPCR primers used to quantify host small intestinal gene expression.

Primer set	Sequence
*β-actin - Forward*	AGCCATGTACGTAGCCATCCA
*β-actin - Reverse*	TGGCGTGAGGGAGAGCATAG
*RegIIIγ - Forward*	TTCCTGTCCTCCATGATCAAAA
*RegIIIγ - Reverse*	CATCCACCTCTGTTGGGTTCA
*MMP7 - Forward*	TTTGATGGGCCAGGGAACACTCTA
*MMP7 - Reverse*	ATGGGTGGCAGCAAACAGGAAGT
*FXR - Forward*	TGGGCTCCGAATCCTCTTAGA
*FXR - Reverse*	TGGTCCTCAAATAAGATCCTTGG
*TGR5 - Forward*	TCCTGTCAGTCTTGGCCTATGA
*TGR5 - Reverse*	GGTGCTGCCCAATGAGATG
*18S - Forward*	ACCGCAGCTAGGAATAATGGA
*18S - Reverse*	GCCTCAGTTCCGAAAACCA

### Crypt isolations and enumerations.

Crypt isolations from mouse small intestine were performed as previously described.^30^ Crypts were then separated from digested tissues using 100 μm cell strainers and counted using light microscopy in 10 μL droplets.

## Histology and immunofluorescence staining

At experiment termination, 3 cm segments of ileum were cut in cross section and fixed in 10% zinc-formalin for 48 hours prior to transfer into 70% ethanol. Ileal villus length and crypt depth (≥10 villus:crypt pairs/mouse) were measured in a blinded manner as previously described using Image J software.^31^ Additional 0.5 cm ileal segments were embedded in an optimum cutting temperature (OCT) media-filled cryomold on dry ice. Embedded tissues were stored at −80°C. Frozen sections (5 μm) were fixed in 1% paraformaldehyde in phosphate-buffered saline and immunostained with mouse anti – ZO-1 (Invitrogen), rabbit anti – claudin-2 (Abcam), or mouse anti – occludin (Invitrogen) followed by Alexa Fluor 488– or Alexa Fluor 594–conjugated secondary antibodies (Invitrogen), along with Hoechst 33,342 (Invitrogen). Stained sections were mounted in ProLong Gold (Invitrogen) and images were captured using a Coolsnap HQ camera (Roper Scientific) mounted on an Axioplan 2 epifluorescence microscope equipped with a Plan-Neofluar 63× NA 1.3 objective (Zeiss) and ET-sputtered single band filter sets (Chroma Technology).^32^ The microscope was controlled using MetaMorph 7 (Molecular Devices). Exposure times were matched between conditions for each antigen, and all post-acquisition processing was standardized for each antigen. Overlays were created using MetaMorph 7 and subsequently rotated using Adobe Photoshop CS6.

### Cell culture experiments.

Mycoplasma-free T84 (CCL-248) and Caco-2 (HTB-37) were procured from the American Type Culture Collection (ATCC). Cells were cultured in DMEM (Gibco) media supplemented with 10% heat inactivated fetal bovine serum (Sigma) and 1% penicillin- streptomycin mix; the latter was eliminated in experiments utilizing *Lactobacillus spp*. Cells were maintained in a water-jacketed incubator in a humidified environment maintained at 37°C with 5% CO2 and checked every two weeks with Mycoplasma test strips (Invivogen). To induce differentiation, 2.5 E5 cells/ml were sown onto Transwell ® (3.0 uM, 12 mm polyester) inserts. For Caco-2 experiments, Transwells were first coated with 30 µg/mL Type I rat tail collagen (Corning 354,236) in 1× DPBS (Life Technologies 14,190,144). Once cells had adhered, transepithelial electrical resistance (TER) was serially measured using an EVOM2 epithelial voltohmmeter (World Precision Instruments) equipped with STX2 hand-held chopstick electrodes. TERs were measured on alternate days for 10–14 days, until their plateau at approximately 1200–2500 Ω/cm^2^; media was changed on alternate days by gently aspirating wells and replenishing with fresh pre-warmed media. TER was measured before and immediately after media changes, and at timepoints indicated on figures. Corrected TER values were calculated as (TER^sample^ – TER^Media^) * Surface Area ( = 1.12 cm^2^). %TER change was calculated as follows: (TER^Corrected_0 hour^ –TER^Corrected_24 hours^)/(TER^Corrected_0 hour^) * 100.

Reagents for TER experiments consisted of basal TYI-S-33 media wherein *Lactobacillus spp*. were cultured as described above,^19^ DMEM with 0.1–10% physiologic bile acids (B8381, Sigma-Aldrich), or DMEM control media. For experiments described in Figure 5 and Supp. Figure 7, serial TERs were measured for a total of 12 or 8 hours respectively at 0.5–12 hour intervals. Data are represented as change from baseline and analyzed using 2-way ANOVA with post hoc Tukey test for multiple comparisons.

## Toxicity, viability, and permeability assays

Toxicity was quantified using CellTox Green (Promega G8742) per manufacturer’s directions. Briefly, at end of time course,1 µL CellTox Green was added into apical compartment of Transwell, and cells were incubated in the incubator in the dark for 30 min, then, 100 µL of apical media was transferred to a 96-well black assay plate to quantify fluorescence (Excitation: 485 nm, Emission: 520 nm) on a ClarioStar Plus Plate Reader (BMG Labtech).

Barrier permeability was assessed as previously reported^33^ using 0.1 mg/ml Lucifer Yellow (LY, Invitrogen Life Technologies, L1177) dissolved in buffer B consisting of Hanks Balanced Salt Solution (HBSS, Gibco Life Technologies 14,175,095) supplemented with 625 mm calcium chloride (Fisher Scientific C614–500), 250 mm magnesium chloride (Honeywell M9272), and 1 M HEPES (Corning 25–060-Cl). The media from apical and basal compartments was removed, washed twice with HBSS, and then replaced with 500 µL LY + buffer B in apical and 1.5 ml buffer B alone in basal compartment. Transwells were incubated for 90 minutes to allow LY diffusion, then 100 µL fluid from each basal compartment was transferred to a 96-well black assay plate, and fluorescence quantified on ClarioStar Plus Plate Reader (Excitation 428 nm, Emission: 536 nm). % LY leak was calculated as follows:

$$\%\mathrm{LYleak}=\frac{\left(\mathrm{Sample}\;\mathrm{Final}\;\mathrm{LY}\;\mathrm{fluorescence}\right)-\left(\mathrm{Blank}\;\mathrm{fluorescence}\right)}{\left(\mathrm{Sample}\;\mathrm{Initial}\;\mathrm{LY}\;\mathrm{fluorescence}\right)-\left(\mathrm{Blank}\;\mathrm{fluorescence}\right)}\times 100$$

### Targeted amino acid and bile acid profiling.

Blood was collected by cardiac puncture at the time of mouse necropsy, and serum was separated using BD serum separator additive microtainer tubes (Catalog# 365967) and centrifugation at 4000 × g RCF for 15 min. Fecal samples also collected at the time of sacrifice were snap-frozen in liquid nitrogen. Supernatants from TYI-S-33 media or DMEM enriched with physiologic bile acids were filter sterilized from bacteria-free or media containing *Lactobacillus* strains in 1 mL aliquots. All samples were stored at − 80°C and shipped on dry ice to the University of Pennsylvania Microbial Culture and Metabolomics Core. The Core performed targeted amino acids quantification using a Waters Acquity uPLC System with a Photodiode Array Detector. Amino acid concentrations are quantified via ultra-performance liquid chromatography (Waters Acquity UPLC system) with an AccQ-Tag Ultra C18 1.7 μm 2.1 × 100 mm column and a photodiode detector array. Analysis was performed using the UPLC AAA H-Class Application Kit (Waters Corporation, Milford, MA) according to manufacturer’s instructions. Standards are run at the beginning and end of each metabolomics run. Quality control checks (blanks and standards) are run every eight samples. Results are rejected if the standards deviate by greater than ±5%. All chemicals and reagents are mass spectrometry grade. Samples without detectable analytes were assigned a value at half the limit of detection, which is 1 nmol/g in stool or 3 uM in plasma. For heatmaps, raw data was transformed to fold change relative to indicated reference groups. Where indicated, amino acids and bile acids were normalized to paired individual mouse intestinal permeability as determined by oral FITC-dextran (4.4 kDa gavage) administered 1.5 hours prior to blood collection, a time point that reflects small intestine localization although some amount of colon permeability could possibly occur.^9^ Simple linear regressions were used to correlate serum levels of bile acid and FITC-dextran.

### Statistics

For all murine experiments, animals were randomized into weight-matched groups at baseline. Due to the requirements to label infectious agents, the investigators were not blinded to allocation during experiments or to growth outcomes. Animal weight data were normalized to baseline absolute weights on day of challenge. For all growth assessments the absolute weights were transformed as percent of initial weight were analyzed using a two-way ANOVA test with repeated measures and Bonferroni posttest analysis for multiple comparisons. For comparisons between only two groups normalized data was compared using two-sided Unpaired t-tests and for non-normalized data two-sided Mann-Whitney U-test was used. The One-Way ANOVA with Holm-Sidak’s or Kruskal-Wallis with Dunn’s multiple comparisons tests were used for multi-group comparisons for normalized and non-normalized data, respectively.

16S amplicon *profiling methodology*: Principal Coordinates Analysis (PCoA) was used to visualize the differences in microbial profiles at genus level between groups with the R package ‘vegan’. Ellipses indicate 95% confidence limits. PERMANOVA test (999 permutations) was used to analyze the differences in genus composition. Differences were considered significant at *p* < 0.05. Differential taxa were analyzed with Mann-Whitney U-test and P-values were adjusted with the Benjamini-Hochberg method to correct for multiple hypotheses testing.

Analyses for all data obtained from mouse models were analyzed with GraphPad Prism version 9.3. Data from all independent experiments are shown in the main figure or in the supplementary figures. Every datapoint indicates measured performed on independent biological replicates as either a single measurement or the mean of technical replicates. Simple linear regressions were used to perform correlation analyses in Figures 4d and Supplemental Figure 8 h.

*Targeted metabolomics methodology*: Serum and fecal amino acid and bile acid profiling data from CD-PBS and PD-PBS groups were filtered considering at least 50% of the valid values. Samples without detectable analyte values were replaced by the limit of detection (LoD) divided by the square root 2 (LoD of 3 uM for serum amino acids, 0.05 μM for serum bile acids, 0.5 nmol/g for fecal bile acids, and 1 nmol/g for fecal amino acids). Variables were selected by discrimination between the pair groups using Sparse Partial Least Squares Discriminant Analysis (sPLS-DA) and Principal Component Analysis (PCA) in MixOmics^34^ on Rstudio. Statistical analyses were performed based on the normality test (Student’s t-test 10% FDR or Wilcoxon rank-sum Test 10% FDR) followed by a 1.5× fold change ratio for each comparison in both fecal and serum samples using “Volcano plot”, “ggplot2”, and “MixOmics” R packages. In the Volcano plot, red dots represent statistically significant overexpressed variables, while blue dots are the underrepresented variables in the first condition in the ratio of the paired comparison (e.g. PD-PBS/CD-PBS, PD-PBS is the first condition in the ratio). Raw data are included as supplemental files.

## Results

### Giardia infection diminishes distinct markers of epithelial cell homeostasis.

**Figure 1.**
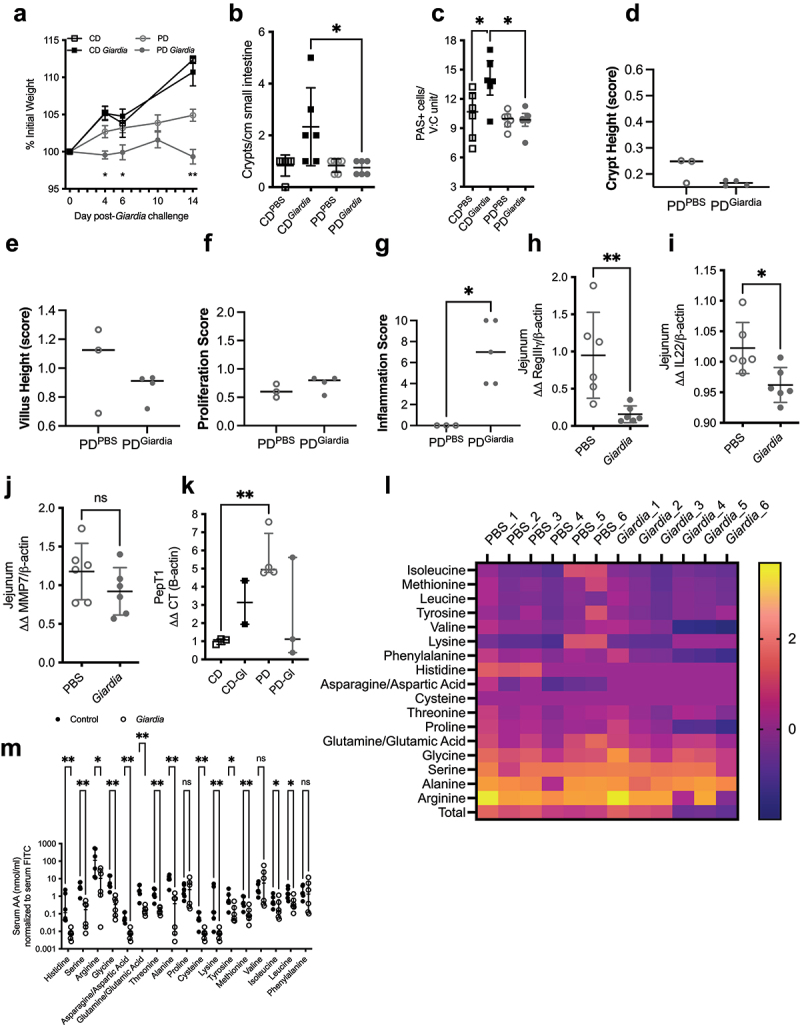
*Giardia* alters markers of intestinal epithelial cell homeostasis that are associated with commensal bacteria functions. A-C) relative gene expression of innate mucosal responses in jejunum of pd-fed PBS controls and 10 days after *giardia* challenge as indicated: (a) *MMP7* (b) *RegIIIγ*, (c) *IL22*. *p < .05 and **p < .01, mann-whitney test, N = 6 per group. (d) Growth as % initial weight of CD or pd-fed PBS controls or *giardia-*challenged mice. Three-week-old specific pathogen free (SPF) mice were acclimated to respective diets for 10 days prior to challenge with 10^5^ *G. lamblia* cysts or PBS. *p < .05, ****p < .0001 two-way ANOVA with Tukey’s post hoc analysis for pd-*giardia* vs. PD (mean ± SEM, N = 6 per group). (e) Enumeration of crypts isolated from the small intestine of mice in figure E. *p<.05, Kruskal-Wallis with Dunn’s test for multiple comparisons as indicated, N = 6 per group (median ± IQR). Morphometry as assessed by scores for crypt (f) and villus (g) heights or overall proliferation (h) does not appreciably differ in pd-fed mice irrespective of *giardia* infection, whereas inflammation scores are higher in *giardia* infected pd-fed mice (*p<.05 by mann-whitney test). (j) Compared to mock, *giardia* infection increases PAS+ staining in villus/crypt (V:C) units in cd-fed mice, a difference that is lost in pd-fed mice. (k) Relative expression of oligopeptide transporter *PepT1* in duodenum of CD or pd-diet fed PBS controls and 10 days after *giardia* challenge. *p<.05, mann-whitney, N = 3 per group. For A-C & K, data are shown as ΔΔ to β-actin housekeeping gene and normalized to PBS controls (median ± IQR). (l) Heatmap representation of ratio of free amino acids measured in serum to fecal compartments [log transformed] in pd-diet fed PBS controls and 10 days after *giardia* challenge. (m) Free amino acids in serum normalized to concomitant measurement of serum FITC as a measure of intestinal permeability. *p<.05, **p<.01, multiple t-tests, Benjamini, Krieger, and yekutielie 2-step method with FDR 5% (median ± IQR, N = 6 per group).

We utilized our previously published specific pathogen free (SPF) model of juvenile protein-deprived mice that develop *Giardia*-enteropathy and growth restriction^19^; in this model, protein deficient diet (PD) fed mice fail to gain body weight compared to mice fed a control diet (CD) and body weight is further attenuated by the presence of *Giardia* (Figure 1a) . Compensatory crypt expansion and absolute numbers of PAS+ cells seen in nourished *Giardia-*challenged mice was not observed in PD-fed *Giardia-*challenged mice (Figure 1b-c respectively) consistent with our previously reported findings.^19^ *Giardia* did not alter crypt or villus height or proliferation score (Figure 1d,e,f) respectively in PD fed mice. Compared with PBS-challenged PD-fed controls, *Giardia*-challenged animals had increased inflammation scores (Figure 1g) but reduced upper small intestinal gene expression of innate responses regulated by RegIIIγ and IL22 in response to Gram-positive bacteria (Figure 1h-i). Interestingly, we did not observe upregulation in matrix metalloprotease 7 (MMP7; Figure 1j) following *Giardia* challenge, which has previously been reported to occur following *Giardia lamblia* infection during adequate nourishment, and important for controlling parasite numbers.^35^ These markers of intestinal mucosal defense responses,^36^ regeneration and repair,^14^ and regulation of transcellular protein transport^37^ are all reportedly modulated by *Lactobacillus spp*.

Reflecting previous findings in malnourished rats,^38^ we found that protein deprivation led to compensatory upregulation of the oligopeptide transporter *PepT1*, which was partially diminished by the presence of *Giardia* (Figure 1k). In accordance with the published literature, we found that protein deficiency significantly altered serum (Supp. Figure S1A-C) and fecal (Supp. Figure S1D-F) pools of free amino acids, regardless of *Giardia* infection status. Serum:fecal ratios of free amino acids in *Giardia-*challenged PD-diet fed mice tended to be lower than PBS controls (Figure 1l, Supp. Figure S1G). We accounted for unregulated paracellular amino acid flux reported to occur with increased gut permeability^9^ by measuring serum FITC-dextran. Adjusting for permeability, *Giardia* challenge significantly reduced serum histidine, serine, arginine, glycine, asparagine/aspartic acid, glutamine/glutamic acid, threonine, alanine, cysteine, lysine, tyrosine, methionine, isoleucine, and leucine (Figure 1m). Levels of proline, valine, and phenylalanine were similar in both groups.

Together these findings suggested that compensatory mucosal and nutrient responses during protein deprivation may be reliant upon functions of critical commensal bacteria but were impeded by *Giardia* colonization.

### Giardia diminishes Lactobacillus and increases Bifidobacterium relative abundances in protein-deprived mice.

**Figure 2.**
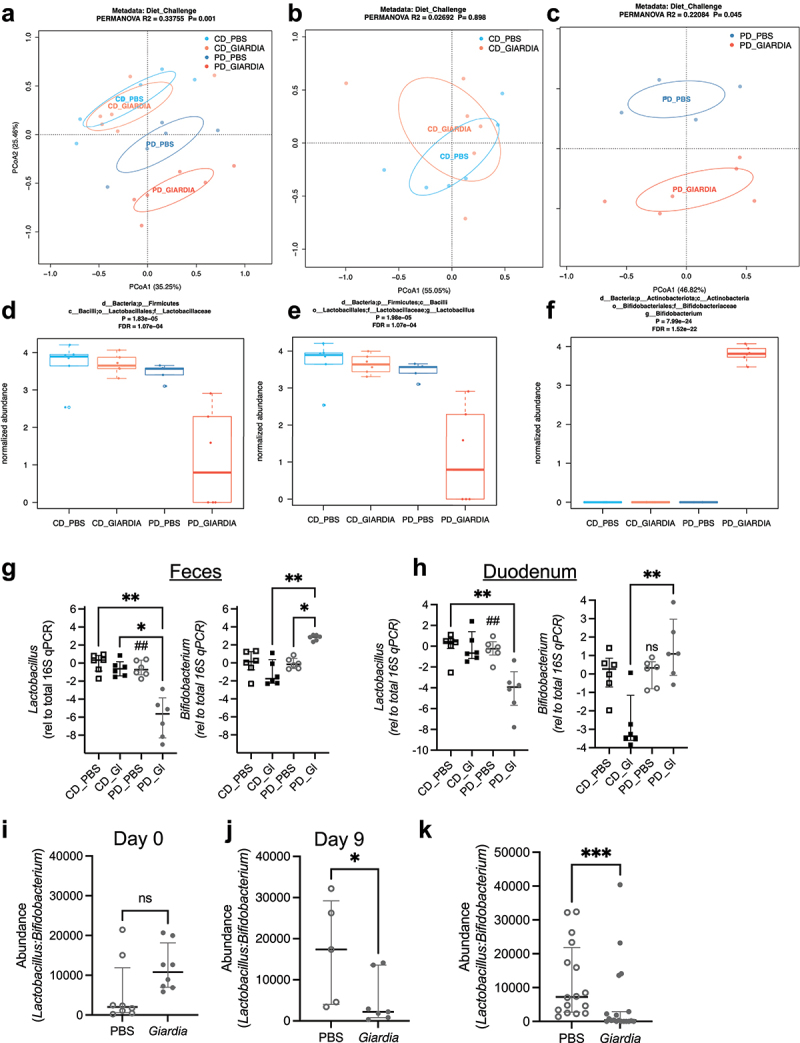
*Giardia* differentially alters *Lactobacillus* and *bifidobacterium* abundances in protein-deprived mice. (a) PCoA plots of fecal taxonomic profiles at genus level from mice fed either a control diet (CD) or an isocaloric protein deficient diet (PD). Samples were collected 10 days after challenge with 10^5^ *G. lamblia* cysts (GIARDIA) or PBS as indicated (N = 6 mice per group, N = 2-3 cages per group). (b) PCoA plot of CD_Giardia and CD_PBS. Fecal taxonomic profiles at genus level were not significantly different between CD_Giardia and CD_PBS (PERMANOVA test: R2 = 0.0269, p = 0.898). (c) PCoA plot of PD_Giardia and PD_PBS. Fecal taxonomic profiles at genus level were significantly different between PD_Giardia and PD_PBS (PERMANOVA test: R2 = 0.221, p = 0.045). Normalized abundances (log10, median ± IQR) of lactobacillaceae (d), *Lactobacillus* (e) and *bifidobacterium* (f). FDR < 0.1, Mann-Whitney U-test. Abundances of *Lactobacillus* and *bifidobacterium* by qPCR from paired fecal (g) and duodenum (h) samples as labeled. Data are represented as relative to total 16S qPCR. *p < .05, **p < .01, Kruskal-Wallis with Dunn’s test for multiple comparisons as indicated, N = 6 per group. (e) Ratios of abundances of *Lactobacillus:bifidobacterium* in fecal samples from PD- fed, PBS- or *giardia-*challenged mice from individual representative experiment: day 0 of *giardia* challenge (i) and day 9 after *giardia* challenge (j). *p < .5 (Mann-Whitney U-test, median ± IQR, N = 5-8 per group. (k) Ratios of relative abundances of *Lactobacillus:bifidobacterium* in fecal samples from pd-diet fed *giardia* challenged or PBS control mice aggregated day 9-11 after *giardia* challenge from three independent experiments, N = 16-19 per group. ****p<.001 (Mann-Whitney U-test, median ± IQR, N = 6 per group).

In the present study, we profiled intestinal bacterial communities using fecal 16S rRNA amplicon sequencing between 9–11 days following cyst challenge, a timepoint at which *Giardia-*colonized PD-fed mice have reproducibly established growth restriction and increased intestinal permeability.^9,10^ No significant differences in alpha diversity were observed between any groups (Supp. Figure S2A-C). In contrast, while protein deficiency drove fecal microbiota community changes, we found that PD-fed mice challenged with *Giardia* had a further alteration in community profiles, compared to those challenged with control (PBS) (Figure 2a-c). In PD-fed mice, the presence of *Giardia* resulted in genus-level alterations in microbiota composition, characterized by a reduction in *Lactobacillus* (Figure 2d-e), and a reciprocal increase in *Bifidobacterium* (Figure 2f). This change was further evident in the taxonomic compositional profiles of these groups (Supp. Fig S2D-H). Targeted qPCR using feces from PD-fed *Giardia*-challenged mice confirmed a > 5-fold reduction in *Lactobacillus* (*p* < 0.01) and a > 2-fold increase in *Bifidobacterium* (*p* < 0.05) relative to total bacteria, as estimated by universal 16S qPCR (Figure 2g). Within the *Giardia* trophozoite-rich duodenum of PD-fed mice, there was a ~ 4-fold decrease in *Lactobacillus* compared with diet matched controls (*p* < 0.01) (Figure 2h). However, *Bifidobacterium* in the duodenum did not appreciably (*p* = 0.132) differ in the presence or absence of *Giardia* (Figure 2h), indicating that protein deficiency may be a primary driver of *Bifidobacterium* abundance.

We individually housed mice and used genus-specific and universal 16S qPCR to quantify baseline *Lactobacillus* and *Bifidobacterium* to account for potential confounders arising from their differences prior to *Giardia* exposure. In this experiment, baseline *Lactobacillus:Bifidobacterium* relative abundances were unequally distributed with slightly higher ratios in the *Giardia-*challenged mice (Figure 2i). However, by 9 days after *Giardia* challenge, *Lactobacillus:Bifidobacterium* relative abundances had significantly decreased compared with PBS-controls (Figure 2j). This decrease was observed in additional age- and diet-matched independent repeats of this experiment, albeit with varying ratios, likely resulting from cohousing (Supp. Figure S2I, J). Aggregating all age- and diet- matched experiments, we found that the presence of *Giardia* reproducibly and significantly diminished *Lactobacillus:Bifidobacterium* ratios within 9–11 days post colonization (Figure 2k).

### Protein deficiency increases primary bile acids, which positively correlates with Giardia-mediated barrier defects

**Figure 3.**
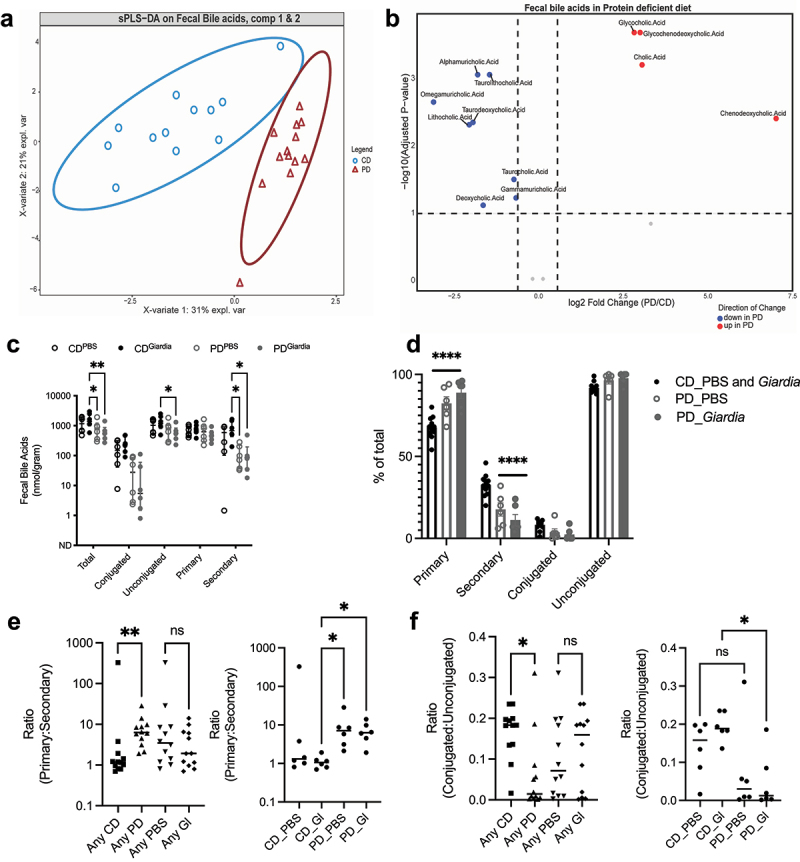
Protein deficiency restricts conversion of intestinal primary to secondary bile acids regardless of *giardia* challenge. (a) sPLS-DA of faecal bile acids from mice fed a protein-deficient diet compared with the control diet (CD). (b) Volcano plot displays the significant bile acids in fecal samples with PD diet compared to control diet (CD). Taurine-conjugated (taurolithocholic acid, taurocholic acid, and taurodeoxycholic acid) and secondary bile acids (alphamuricholic acid, omegamuricholic acid, gammamuricholic acd, lithocholic acid, and deoxycholic acid) were identified as underrepresented in protein deficient diet (blue dots). In contrast, glycine-conjugated (glycocholic acid, glycochenodeoxycholic acid) and primary bile acids (cholic acid and chenodeoxycholic acid) were overrepresented in protein deficient diet (red dots) ((wilcoxon rank-sum test, 10% FDR). (c) Comparisons between total, conjugated, unconjugated, primary and secondary bile acids. *p < .05, **p < .01, two-way ANOVA with Tukey’s posttest analysis for multiple comparisons (median ± IQR, N = 6 per group). (d) Percentage of total bile acids represented by primary, secondary, conjugated and unconjugated types. *p < .001 for PD_PBS or PD_*Giardia* vs. CD_PBS and *giardia* groups combined. Two-way ANOVA with Tukey’s posttest analysis for multiple comparisons (median ± IQR = 6-12 per group). (e) Ratios of primary:secondary bile acids in aggregate groups by dietary or *giardia* exposure (left) and individual groups (right). ****p < .05, *p < .01 for indicated groups. Kruskal-Wallis with Dunn’s test for multiple comparisons as indicated, (median ± IQR, N = 6 per group). (f) Ratios of conjugated:unconjugated bile acids in aggregate groups by dietary or *giardia* exposure (left) and individual groups (right). *p < .05 for indicated groups. Kruskal-Wallis with Dunn’s test for multiple comparisons as indicated, (median ± IQR, N = 6 per group).

A primary metabolic function of several commensal *Lactobacillus* species is regulation of bile acid homeostasis through the expression of bile salt hydrolases (*bsh*) that deconjugate glyco- or tauro-conjugated primary bile acids.^39^ Allochthonous *Lactobacillus* strains diminish transient *Giardia* infection in murine models through this deconjugation activity, increasing the pool of deconjugated bile salts,^40,^^41,42^. In a similar vein, mice persistently infected with *G. lamblia* had decreased amounts of taurine-conjugated bile acids, and increased levels of the corresponding unconjugated bile acids.^43^ To determine if altered *Lactobacillus:Bifidobacterium* ratios contributed to disrupted bile acid homeostasis during protein deprivation, we profiled fecal and serum bile acids at 10/11 days post-*Giardia* challenge in PD-fed and adequately nourished mice (20 days total on either PD or regular chow). Fecal bile acid profiles were largely diet-dependent (Figure 3a-c), without a remarkable influence of *Giardia* challenge (Supp. Figure S3A, B). Regardless of *Giardia* status, the PD-diet led to an increase of primary bile acids relative to secondary bile acids (Figure 3d-f, Supp. Figure S3B). Comparisons between conjugated and unconjugated bile acids showed more variability between PD-diet fed mice (Figures 3c-d), but overall diminished amounts of both glyco- and tauro-conjugated bile acids in PD- fed mice indicated preserved bacterial *bsh* activity (Figure 3c-d).

**Figure 4.**
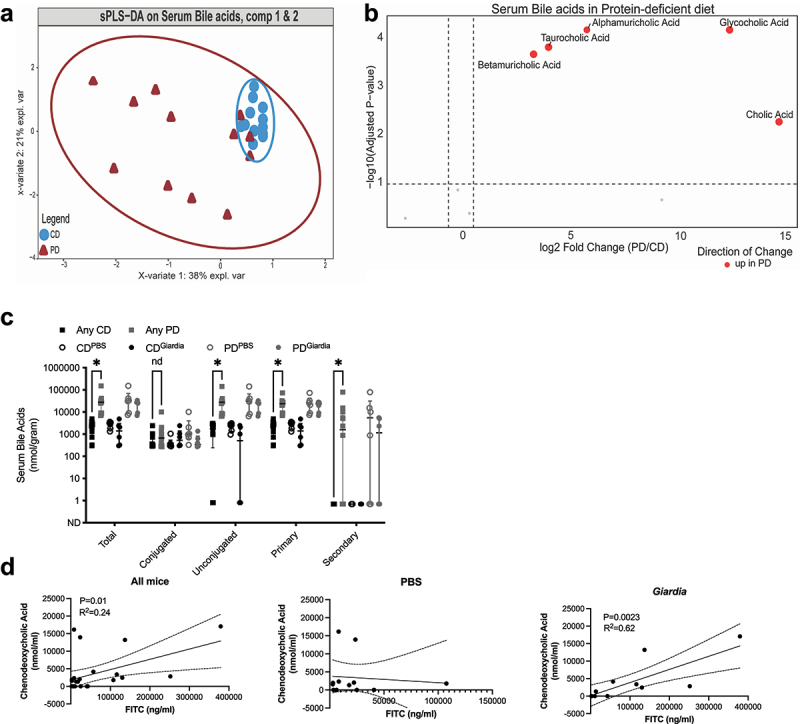
Protein deficiency increases circulating bile acids including chenodeoxycholic acid which correlates with severity of intestinal permeability in *giardia* challenged mice. (a) sPLS-DA analysis of serum bile acids in mice fed a protein-deficient diet (PD) compared to mice fed a 20% protein diet (CD). (b): volcano plot showing the significant abundance of serum bile acids in PD diet (wilcoxon rank-sum test, 10% FDR) with increased levels of glycine-conjugated bile acids (glycocholic acid), taurine-conjugated bile acids (taurocholic acid), and secondary bile acids (alphamuricholic acid and betamuricholic acid), (red dots in the volcano plot). (c) Comparisons between total, conjugated, unconjugated, primary and secondary bile acids. *p < .05, multiple t-tests, Benjamini, Krieger, and yekutielie 2-step method with FDR 5% (median ± IQR, N = 6 per group). (d) Correlation between serum FITC (ng/mL) and chenodeoxycholic acid (nmol/mL) in pd-diet fed mice (left, all mice; middle, only PBS controls; right only *giardia* challenged mice). Simple linear regression (N = 6 per group), R^2^ = 0.009238.

In our model, differences in serum bile acid signatures between groups were also primarily driven by diet (Figure 4a-b). PD-diet increased total bile acids, with minimal impact of *Giardia* even in PD-fed mice (Supp. Figure S4A, B). Additionally, ileal expression of *Fxr* or *Tgr5* bile acid homeostasis regulators were comparable between groups (Supp. Figure S4C). Although alterations in serum BA profiles are primarily driven by diet (Figure 4a-c), serum chenodeoxycholic acid concentrations were significantly and positively correlated with FITC-dextran, a measure of intestinal permeability, in PD-fed *Giardia*-infected mice, indicating a positive association with *Giardia-*induced barrier defects (Figure 4d). This relationship was absent in PD-fed PBS-challenged controls. These data suggest that although *Giardia* does not change global intestinal and serum bile acid profiles in our model, *Giardia* colonization may have altered host susceptibility to bile acid-induced epithelial barrier defects occurring during undernutrition.^44,45^

### Bile acid-induced barrier disruption is rescued by bsh-producing L. plantarum

**Figure 5.**
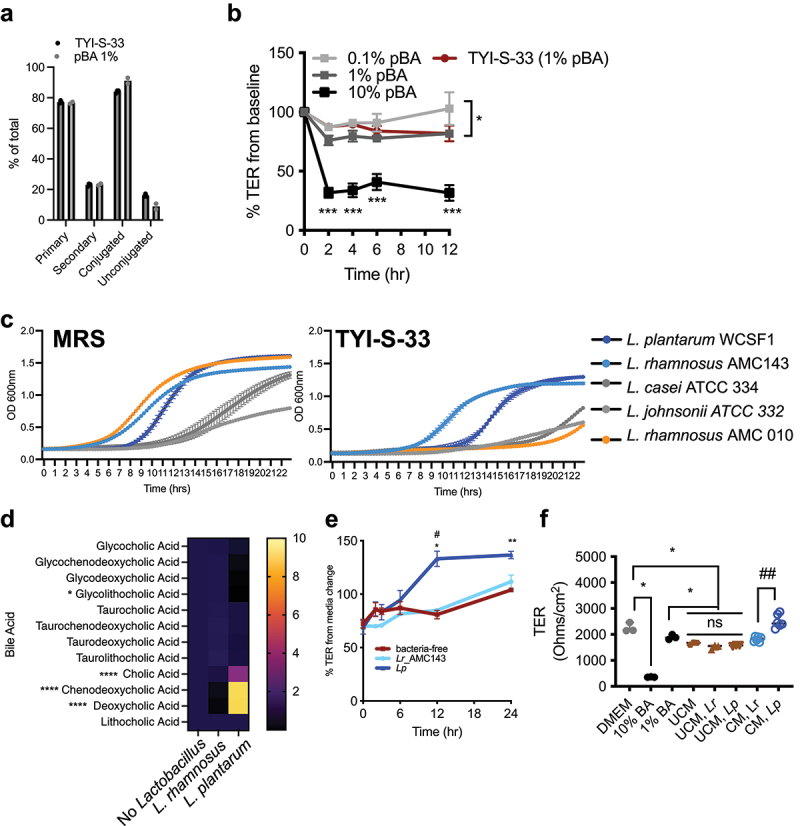
Physiological bile acids differentially support *lactobacillus spp*. growth and protective functions of *lactobacillus* on bile-acid induced intestinal epithelial cell barrier injury are strain specific. (a) Compositional analysis by percentage of total bile acids represented by primary, secondary, conjugated and unconjugated types present in physiological bile acids mixture when dissolved in DMEM (pBA 1%) or protozoan media (TYI-S-33). (b) Transepithelial cell electrical resistance (TER) as % change from baseline in *T*-84 monolayers exposed do different concentrations of pBA in DMEM (0.1, 1.0 and 10%) or TYI-S-33 media containing 0.1% pBA. *p < .05, two-way ANOVA for 0.1% pBA vs either 1% pBA or TYI-S-33 media, **p < .001. Two-way ANOVA with Tukey’s posttest analysis for multiple comparisons for 10% pBA vs 0.1% pBA (median ± IQR, N = 3 wells group). (c) Growth curves of different *lactobacillus* strains in MRS media (left) or TYI-S-33 (right). Shown are means of at least 6 technical replicates from each strain. For *L. plantarum* and *L*. rhamnosus_AMC143, curves are the mean of two separate biological replicates with at least technical six replicates each (d) heat map of bile acid profiles in TYI-S-33 media after 24 hours of growth of *lp* or *Lr*_AMC143 relative to baseline fresh media. (e) TER as % change from baseline in *T*-84 monolayers exposed to TYI-S-33 with either log-phase 10^6^ *lp* or *Lr*_AMC143 or bacteria-free media. * p < 0.05, ** p < 0.01 for bacteria-free vs *lp* and # p < 0.05 for *lp* vs *Lr*_AMC143, two-way ANOVA with Tukey’s posttest analysis for multiple comparisons (mean ± IQR, N = 3 per group). (f) Baseline TER at 30 minutes after media change from DMEM to TYI-S-33 alone, or TYI-S-33 containing log-phase 10^6^ *lp* or *Lr*_AMC143, or filter-sterilized conditioned TYI-S-33 wherein *lp* or *Lr*_AMC143 were cultured for 24 hours, as indicated. *p < .05 for DMEM vs 10% BA (Kruskal-Wallis with Dunn’s correction for multiple comparisons DMEM vs 10% BA or 1% BA), ****p < .05 for DMEM or 1% BA vs UCM and UCM^*Lr*^ and UCM^*Lp*^ (Kruskal-Wallis with Dunn’s test for multiple comparisons DMEM vs 1% BA, UCM, UCM^*Lr*^, and UCM^*Lp*^). *p < .01 for CM^*Lr*^ vs CM^*Lp*^ (mann-whitney U-test) (median ± IQR, N = 3-6 per group).

Bile acids may have either homeostatic or toxic consequences on epithelial cells^46^ depending on the concentration and hydrophobicity of bile acid species that are present. We directly tested how a physiologically relevant mixture of bile acids perturbs intestinal epithelial cell permeability using the T84 cell line. Confluent cell monolayers were grown until their transepithelial electrical resistance (TER) plateaued, indicative of a robust impermeant barrier. At this point, monolayers were exposed to a mixture of physiological bile acids dissolved in DMEM (termed pBA), or in *Giardia*-trophozoite supportive TYI-S-33 media; both contain 1%(w/v) bile acids in the proportions indicated in Figure 5a and Supp. Figure 8A, B (and tabulated in Supp. Table S1). Application of bile acids dissolved in either pBA or TYI-S-33 media resulted in immediate (within 2 h) and sustained loss of TER indicative of a loss of barrier function (Figure 5b). TER reduction occurred in a dose-dependent manner and had not recovered up to 12 hours following bile acid exposure.

We next examined whether the reported barrier-protective properties of *Lactobacillus* strains could protect from bile acid-induced disruptions in epithelial barrier integrity.^13^ We first identified *Lactobacillus spp*. strains that were bile tolerant, as defined by achieving stationary phase growth in <24 hours in 1% BA-containing TYI-S-33 media in aerobic conditions (Figure 5c). Two strains, *L. plantarum* WCSF-1 and *L. rhamnosus*^AMC143^ met these criteria. Both reach stationary phase at approximately similar times when grown in either MRS or TYI-S-33 media (Supp. Figure S5). Moreover, consistent with known differences in their functional *bsh* activity^21,47^ both strains differed in their ability to deconjugate bile acids *in vitro* (Figure 5d; Supp. Figure S6A). We also assessed bile tolerance and deconjugation activity of *Lactobacilli spp*. strains under anerobic conditions (Supp. Figure S6A, B respectively). We find that compared to *L. rhamnosus*^AMC143^, *L. plantarum*^WCSF-1^ has a higher growth in media supplemented with 0.2% TCA, GCA, TDCA or GDCA. *L. plantarum*^WCSF-1^ also deconjugated each of these bile acids into their respective unconjugated forms, as evidenced by precipitates in solid media (Supp. Figure S6B). Incubation with *bsh-*expressing *L. plantarum*^WCSF-1^ but not *L. rhamnosus*^AMC143^ protected T84 monolayers from pBA-induced barrier injury. The amelioration of bile acid-induced barrier defects occurred within 6 hours of incubation with viable *L. plantarum*^WCSF-1^ (Figure 5e). Barrier protective effects of *L. plantarum*^WCSF-1^ were also confirmed using the Caco-2 cell line by serial measurements of TER and quantifying the translocation of a fluorescent reporter lucifer yellow from the apical to basolateral compartment at the terminal time point. By these two readouts, we confirmed that *L. plantarum*^WCSF-1^ maintained integrity of Caco-2 monolayers in contrast to TYI-S-33 or *L. rhamnosus*^AMC143^ (Supp. Figure S7A, B respectively). Furthermore, this protective effect was also transferrable through application of filter-sterilized TYI-S-33 conditioned media (CM) within which *L. plantarum*^WCSF-1^ was grown overnight (Figure 5f). Importantly, barrier protection was not observed with unconditioned media (UCM), or conditioned media from *L. rhamnosus*^AMC143^.

### Giardia directly antagonizes growth-promoting effects of commensal Lactobacillus strains in protein-deficient mice.

**Figure 6.**
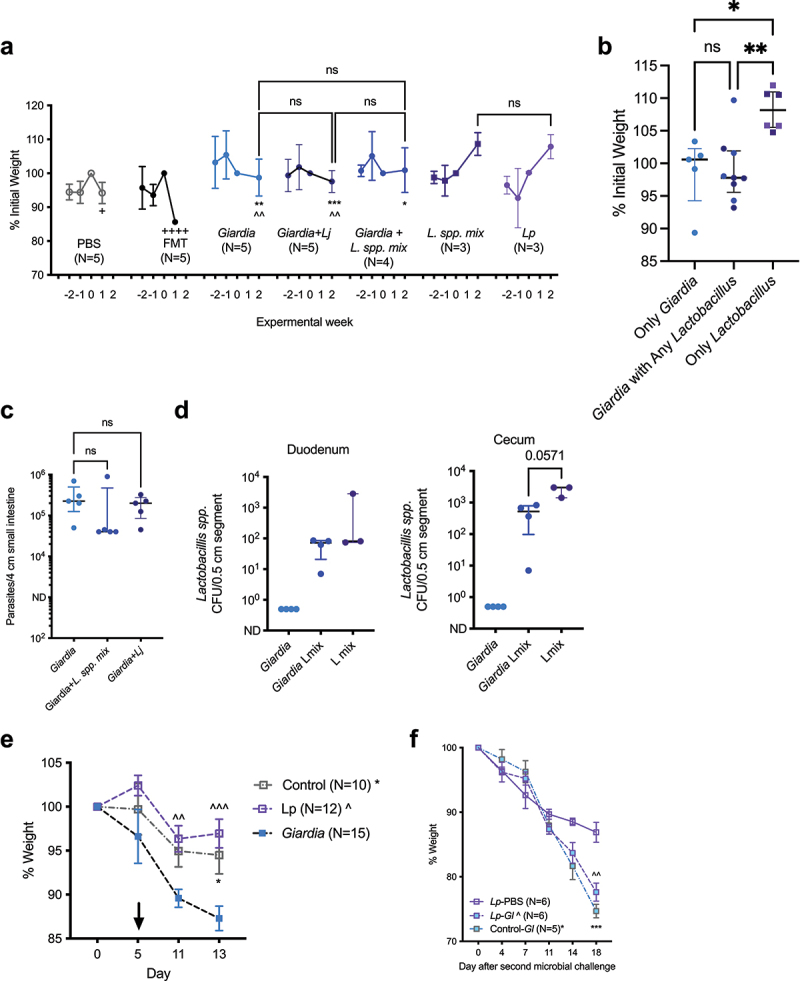
*Giardia* directly antagonizes growth-promoting *lactobacillus spp*. in gnotobiotic mouse models of protein malnutrition. (a) Growth as % weight on the day of microbial challenge (0), beginning two weeks prior to and through 1 after challenge with PBS or fecal microbiota from SPF protein deficient mice (FMT), and two weeks after challenge with either 10^4^ axenic *G. lamblia* cysts or 10^6^ *lactobacillus* strains alone or together as indicated. *L. spp. mix* = 10^6^ each of *lj, lp, Lr_*AMC010, *Lr_*AMC143, and *L. casei*. Three- to six-week-old germ-free (GF) mice were fed a protein deficient diet for the two weeks prior to transfer from isolators and oral gavage with indicated microbial challenge. Experiments were performed sequentially due to limited availability of age-matched GF mice. ^++++^*p* < 0.0001 for PBS vs FMT (week 0-1); ^^^^*p* < 0.01 *lp* vs *giardia* or *Giardia*+*Lj* (week 0-2); *p < .05 for *L. spp. mix* vs *Giardia+ L. spp. mix*, **p < .01 for *L. spp. mix vs giardia*, *p < .001 for *L. spp. mix* vs *Giardia*+*Lj*, two-way ANOVA with Tukey’s posttest analysis for multiple comparisons (mean ± SEM, N = 3-5 per group). (b) Growth as % initial weight through two weeks aggregated by presence or absence of *giardia* challenge or any *lactobacillus* strain. ****p < .05, *p < .01 (Kruskal-Wallis with Dunn’s correction for multiple comparisons, median ± IQR, N = 5-9 per group). (c) *giardia* trophozoites in the small intestine in mono-associated mice and mice co-colonized with *giardia* and *lactobacillus* strains recovered two weeks after colonization (Kruskal-Wallis with Dunn’s test for multiple comparisons, median ± IQR, N = 5 per group). (d) *lactobacillus* colony recovery two weeks post colonization on MRS plates from small intestine and cecal tissues in *giardia* mono-associated mice, mice co-colonized with *Giardia*+*L. spp. mix*, and mice colonized with *L. spp. mix* without *giardia* (Kruskal-Wallis with Dunn’s test for multiple comparisons, median ± IQR, N = 3-5 per group). (e) Growth of 8-16 week old GF *Rag2*^*-/-*^ mice as % initial weight following challenge with *giardia* or *lp* alone, or no microbial challenge (control). Mice were transitioned from control to protein deficient diet on day 5 as indicated by the arrow. ^^P < 0.01, ^^^P < 0.001 *lp* vs *giardia* and *p<.05 control vs *Giardia*; two-way ANOVA with Tukey’s posttest analysis for multiple comparisons (mean ± SEM, N = 10-15 per group. (f) Growth of a subset of control (N = 5) or *lp-*mono-associated (N = 12) *Rag2*^*-/-*^ mice for 13 days as % change following secondary challenge with either *giardia* or PBS as indicated. Mice remained on a protein deficient diet. ^^P < 0.01 for *lp-gl* vs *lp*-pbs and *p < .001 for control-*gl* vs *lp*-pbs. Two-way ANOVA with Tukey’s posttest analysis for multiple comparisons (mean ± SEM, N = 5-6 per group).

Mono-colonization with select *L. plantarum* strains can maintain infant mouse growth despite undernutrition.^12^ To directly test the interactions between *Lactobacillus spp*. and *Giardia* during undernutrition, we used gnotobiotic techniques to selectively colonize germ-free (GF) mice fed a PD diet. Because 16S rRNA amplicon sequencing could not definitively confirm *Lactobacillus* species-level assignments, we designed a consortium of *Lactobacillus spp*. mixture (*L. spp. mix*) using distinct published beneficial functions: *L. plantarum* known to improve intestinal barrier and promote growth; *L. casei* reported to diminish *Giardia* severity in a murine model^41,42^ and promoting expression of the intestinal transport protein PEPT1^37^;* L. johnsonii* a known antagonist of *Giardia* replication^17^; *L. rhamnosus* that regulates host responses to *Giardia* in a different murine model.^16^ Selection of different consortium members may have altered the specific conclusion. In these experiments, GF mice were weaned onto the PD diet in gnotobiotic isolators, prior to transfer into individually ventilated cages in the gnotobiotic barrier facility. First, compared with PBS-challenged GF controls, GF mice conventionalized with fecal intestinal microbiota (FMT) from PD-diet fed *Giardia*-free SPF mice demonstrated rapid weight loss and moribund condition requiring termination of the experiment by 1-week post-FMT (Figure 6a). Whereas *Giardia* monoassociation had no direct effect on weight gain through up to 2 weeks post-challenge, selective colonization with *L. spp*. mix or *L. plantarum* alone promoted similar levels of weight gain with similar kinetics. The presence of *Giardia* antagonized the growth benefit from *L. spp. mix*. Fecal bile acid profiling confirmed the presence of functional bsh activity in *Giardia* + *L. spp. mix* co-colonized mice that was like *L. plantarum* alone, and not the result of *Giardia* activity, consistent with the current understanding that *Giardia* is incapable of bile salt deconjugation (Supp. Figure 8C). Consequently, *Lactobacillus* significantly promoted weight gain during protein deprivation only when *Giardia* was absent (Figure 6b). *Giardia* colonized the SI with similar efficiency in both mono-association and in combination with *L. spp. mix* (Figure 6c). Although *L. johnsonii* has been shown to expedite *Giardia* clearance in nourished mice,^17^ this strain was not recovered from mice co-challenged with *L. johnsonii* in combination with *Giardia* during protein deficiency (Supp. Figure 8D). *Lactobacillus spp*. duodenal and cecal colonization density as determined by colony counts on MRS agar were also similar regardless of *Giardia* exposure in *L. mix spp*. challenged mice (Figure 6d).

Finally, we examined the reciprocal interactions between *Giardia* and barrier-protective and growth promoting *L. plantarum* WCSF-1 (*Lp*) in our model of more severe protein-malnutrition in immunodeficient mice (*Rag2*^−/−^). In these studies, we selectively monoassociated GF immunodeficient mice for 5 days with either *Lp* or *Giardia* for 5 days; then, all mice were transitioned to the PD diet. Whereas *Giardia* mono-association led to expected weight loss through two weeks post-challenge,^9^ *Lp-*monoassociated mice maintained body weight, which was slightly higher than control, germ-free PD-diet fed *Rag2*^−/−^ mice (Figure 6e). After 13 days of colonization, we then performed a heterologous challenge with the second microbe, i.e. *Lp*-monoassocoiated mice were challenged with *Giardia*, and *Giardia*-monoassociated mice were challenged with *Lp*. When *Lp* was introduced into *Giardia*-colonized mice, there was a slight rescue in body weight compared to mice colonized with *Giardia* alone; PD-fed immunodeficient mice colonized with *Lp* alone had the most stable body weight (Figure 6f). Furthermore, progressive weight loss in PD-fed *Giardia-*monoassociated immunodeficient mice could not be rescued by introducing *Lp* (Supp. Fig 8E). Regardless of the order in which *Giardia* was introduced, it colonized the SI to similar levels (Supp. Fig 8F); *Lp* had no effect on trophozoite density within the duodenum (Supplemental Figure 8F). Similarly, *Lp* colony counts in both duodenum and colon were comparable regardless of the order in which *Lp* was introduced, i.e. prior to or after *Giardia* colonization (Supplemental Figure 8D, G). This indicates that neither microbe exerts antimicrobial action per se against the primary colonizer. However, *Giardia* introduced in either order shifted the biogeographic distribution of *Lp* from the duodenum into the colon (Supplemental Figure 8 G, H). This displacement may have prevented *Lp* from exerting growth-promoting functions in protein-undernourished mice. Our findings reveal that interactions between *Giardia* and co-existing intestinal microbes may have highly relevant impacts on host health not discernible by conventional microbiome or metabolome profiling.

## Discussion

In the small intestine, metabolic and nutrient homeostasis is coupled with functions of the local microbial community, whose disruption can result in a loss of mucosal immunoregulation, aberrant nutrient absorption, and perturbed metabolism,^18^ sometimes with exacerbated inflammation.^5^ Emphasis on microbe-mediated contributions to these pathologies have largely focused on the presence of pathogenic invaders, or a detrimental shift in the balance of resident microbes that enhance expression of pathobiont properties. To date, it is unclear how the loss of commensal microbe functions that provide beneficial physiological and homeostatic support contributes to observed pathologies. Here, we report a new gnotobiotic approach to elucidate the potential consequences of displacement of beneficial small intestinal microbes by an environmental invader, *Giardia*. The presence of *Giardia* within the murine host diminished mucosal homeostasis, impaired host growth, and most importantly, counteracted the beneficial, growth-promoting effects exerted by *Lactobacillus spp*. in an undernourished, protein-deprived host. Our work addresses previously reported discrepancies in human studies highlighted by Fekete et al.^48^ and us^49^ that find diametrically opposed changes in the relative abundance of *Lactobacillus spp*. in the presence of *Giardia* natural infection in humans. We find that beyond compositional changes, *Giardia* alters the functional output of the resident microbiota which is arguably more consequential for the host.

We and others have previously reported using murine models that *Giardia* induces compositional or functional shifts in resident intestinal microbiota by altering microbe-host co-metabolic activity, increased virulence traits of pathobionts, and/or co-infecting bacteria.^10,50–52^ Here, we first demonstrate that *Giardia* infection exacerbates the already disrupted protein absorption occurring in protein malnourishment thus further reducing circulating amino acids. Although time-resolved studies incorporating labeled amino acids would enhance the accuracy of our findings, they are outside the scope of the present work. We also report that the presence of *Giardia* may also diminish the abundance, intestinal distribution, and/or physiological functions of autochthonous lactic acid bacteria, even if other shifts in microbial community profiles were not observed by 16S rRNA amplicon sequencing. Importantly, our gnotobiotic experiments indicated that the loss of beneficial functions of *Lactobacillus spp*. during *Giardia* co-colonization were not simply due to parasite-induced competitive exclusion of all *Lactobacillus* strains – only modest reductions in colony counts were observed in the upper small intestine of co-colonized mice. It is therefore more likely that *Giardia* alters the microenvironment leading to changes in commensal bacterial adaptations. In this way *Giardia* may magnify the uncoupling of *Lactobacillus* from mucosal-associated micro-niches as has been reported in undernourished mice evidenced by diminished *Lactobacillus-*specific IgA.^18^ *Giardia* has also been shown to alter expression and downstream functions of nonpathogenic *E. coli* strains.^50^ Alternatively, as we have previously published, *Giardia-*mediated alterations in host responses to bacterial ligands^53^ may also dampen signaling pathways necessary for host responses to commensal bacteria.

Our work revealed significant alteration of *Lactobacillus spp*. in our *Giardia* model. *Lactobacillus* are a closely studied genus of high interest in the field of probiotic interventions for malnutrition, and they have well-defined bile acid metabolizing properties discernible at the strain level.^39,40^ It is known that bile enriched for conjugated bile salts with higher hydrophobicity^54^ and phosphatidylcholine^43^ promotes *Giardia* replication. In contrast, *Lactobacillus spp*. that deconjugate bile acids inhibit *Giardia* growth *in vitro* ^55^ and accelerate *Giardia* clearance in nourished hosts.^17,56,57^ Considering these prior observations, we were surprised to find that *Lactobacillus spp*. inhibition of *Giardia* colonization was impeded in the context of a protein-deficient host. On the surface, this finding is counter to the improved anthropometric and histological responses reported in undernourished BALB/c *Giardia* models treated with *L. casei*^MTCC 1423.58^ However, closer examination of the growth trajectories reported therein (accounting for differences in baseline weights of mice) reveals only modest benefits of *L. casei* on growth during malnutrition, or as an adjuvant to renourishment.^42,58^ The presence of *Giardia* further limits this benefit, and moreover, diminishes recovery of *L. casei* from fecal specimens by up to 4-logs, even if *Giardia* fecal cyst shedding was only modestly decreased. Despite the robust growth advantage conferred by the *Lactobacillus spp*. used in our selectively colonized, protein undernourished murine model, the additional presence of *Giardia* significantly antagonized this benefit. Further studies are required to determine whether *Giardia* similarly diminishes specific autochthonous *Lactobacillus spp*. strains in humans, especially in a diet-dependent manner. It will also be important to determine if *Giardia* similarly interferes with *Lactobacillus spp*. probiotics used for undernourished children. Indeed, current limitations in understanding of these principles may contribute to lackluster results from *Lactobacillus* probiotic interventions for undernutrition.^59^ Based on the 16S rRNA amplicon sequencing data from our model, and a lack of robust colonization competition between *Giardia* and *Lactobacillus ssp*. throughout the gut, we predict that resolving these complex interactions in human studies will require high-resolution approaches for accurate taxonomic characterization of microbial communities. Moreover, metagenomic and transcriptomic characterization of diverse intestinal microenvironments will aid in uncovering the influence of *Giardia* on the functional and spatial localization of resident bacteria within micro-niches. These approaches may help resolve the mixed data generated using amplicon sequencing between children with or without *Giardia* infection.^60,61^ Additionally, it is important to consider that the important interactions between *Giardia* and commensal bacteria may overlap in function but track to different taxonomic designations in humans compared with rodent models, further highlighting the importance of future functional studies.

*Giardia* associates with increased small intestinal permeability in human cohorts and in our murine model.^9^ While *Giardia* may have direct effects on intestinal epithelial cell (IEC) barrier function through alterations in MLCK and ZO-1,^62^ our data suggests that perturbations in the small intestinal microenvironment of protein undernutrition, such as overrepresentation of primary bile acids, may directly harm IECs when beneficial commensal functions are lost. Whereas a comprehensive study of indigenous mouse *bsh*-producing lactic acid bacteria, and their specific *bsh* activities is beyond the scope of this study, we did identify variation in strain-level properties of *Lactobacillus* that are important determinants in recovery from bile-acid inducible injury of intestinal epithelial cells. Physiological concentrations of primary bile acids caused *in vitro* barrier defects that were mitigated by *L. plantarum*^WCSF-1^ which encodes four functional bile salt hydrolase genes. In contrast, *L. rhamnosu*s^AMC143^, which encodes one non-expressed bile salt hydrolase gene^21^ was unable to ameliorate bile-induced barrier defects. Due to technical challenges in facile manipulation of their *bsh* genes, it remains unknown which one or more of the four active *bsh* enzymes of *L. plantarum*^WCSF-1^ impart barrier protective functions; it is also unclear if the presence of *bsh* activity was incidental to other potential IEC-supportive properties present in *L. plantarum*^WCSF-1^ that are absent in *L. rhamnosus*^AMC143^. We note that others have also found strain-selectivity in health-promoting *Lactobacillus* during undernutrition: for example, intraspecies differences were seen in the linear growth benefit resulting from *L. plantarum*^WJL^ but not *L. plantarum*^NIZ02877 12^. Future studies to pinpoint the specific mechanisms through which *L. plantarum* and other *Lactobacillus spp*. promote IEC barrier integrity, either related to specific *bsh* or non-*bsh* activities, and bile acid regulation are relevant not only for *Giardia* enteropathy, but likely for additional pathologies of the upper small intestine, e.g. disrupted bile acid profiles in children with environmental enteric dysfunction (EED).^45^ Our work highlights the utility of gnotobiotic techniques to determine which colonizing species of *Lactobacillus* and *Bifidobacterium* are most relevant for *Giardia* interactions.

In conclusion, we investigated how small intestinal invaders interact with resident microbiota in the undernourished gut. We report that outcomes of mucosal and nutrient dysregulation and impaired host growth during *Giardia* infection may not only be due the presence of a pathogen but the absence of beneficial microbial functions that promote host health. While ongoing studies examining pathogen virulence factors and interventions to neutralize them remain important, our work underscores the importance of considering the entire scope of functional consequences resulting from disrupted small intestinal ecology in undernourished children. Further elucidating the conditions that facilitate colonization of beneficial microbiota in the small intestine and moreover sustain their functional resilience is likely a necessary consideration when devising optimized therapies and novel interventions that not only eliminate the harm but also promote health.

## Supplementary Material

Supplemental Material

## Data Availability

Data associated with this manuscript are available by contacting the corresponding authors. 16s rRNA amplicon sequencing data are accessible using Project ID 1,068,627 on the NIH BioProject Repository.
