# Supplementary figures and images for: Giardia antagonizes beneficial functions of indigenous and therapeutic intestinal bacteria during protein deficiency
Source: Gut Microbes. 2024 Nov 5;16(1):2421623. doi: 10.1080/19490976.2024.2421623 (PMC11542603; doi:10.1080/19490976.2024.2421623)

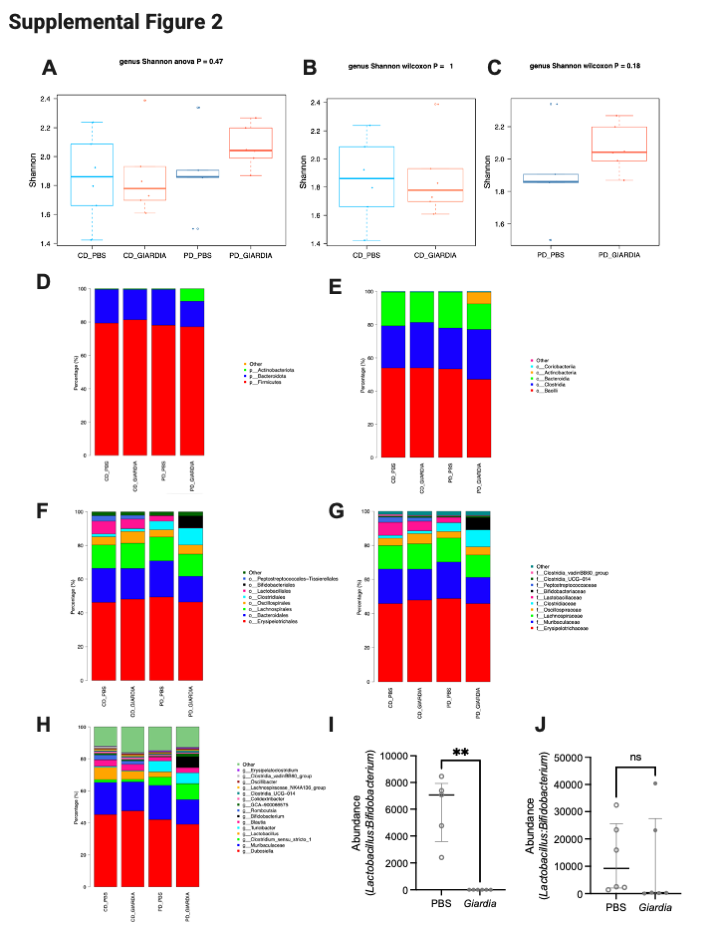

Supplement: Supplemental Material [file KGMI_A_2421623_SM1877.zip › SuppFig2.tiff]

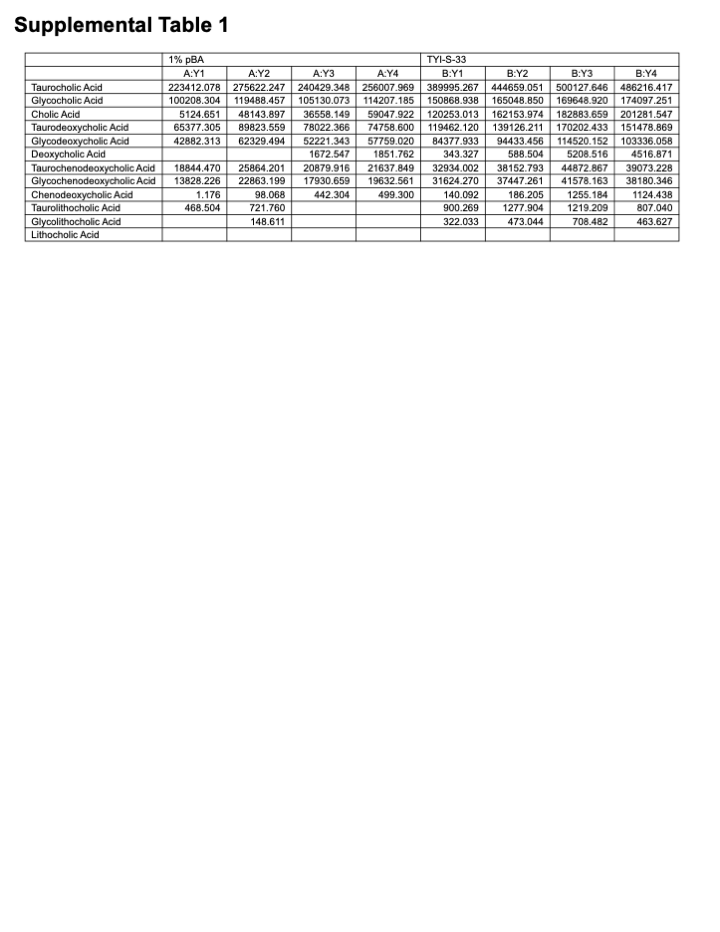

Supplement: Supplemental Material [file KGMI_A_2421623_SM1877.zip › SuppTable1.tiff]

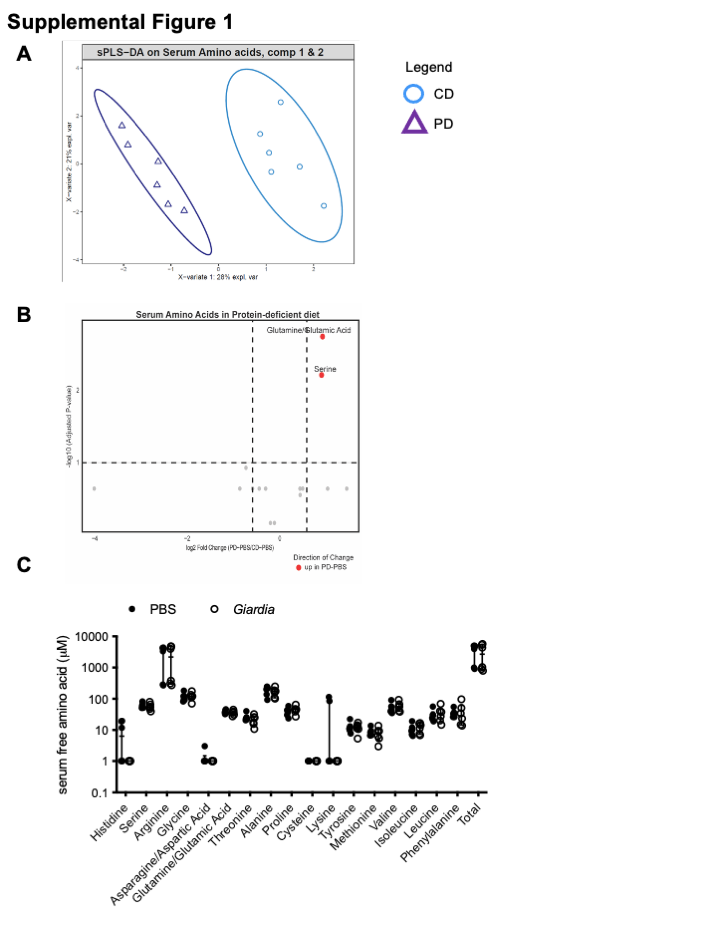

Supplement: Supplemental Material [file KGMI_A_2421623_SM1877.zip › SuppFig1a-c.tiff]

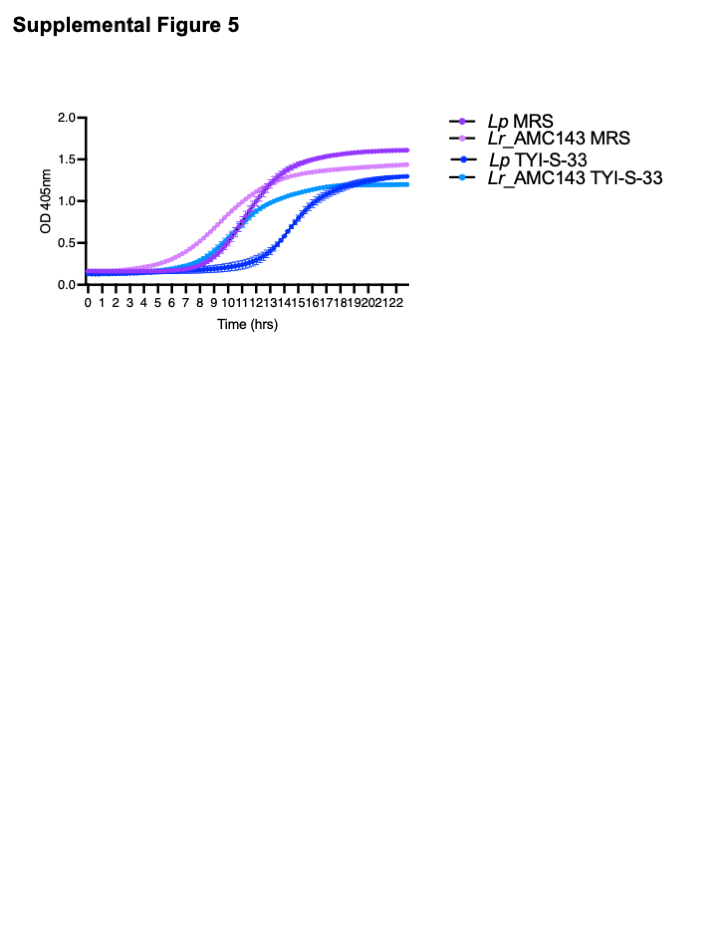

Supplement: Supplemental Material [file KGMI_A_2421623_SM1877.zip › SuppFig5.tiff]

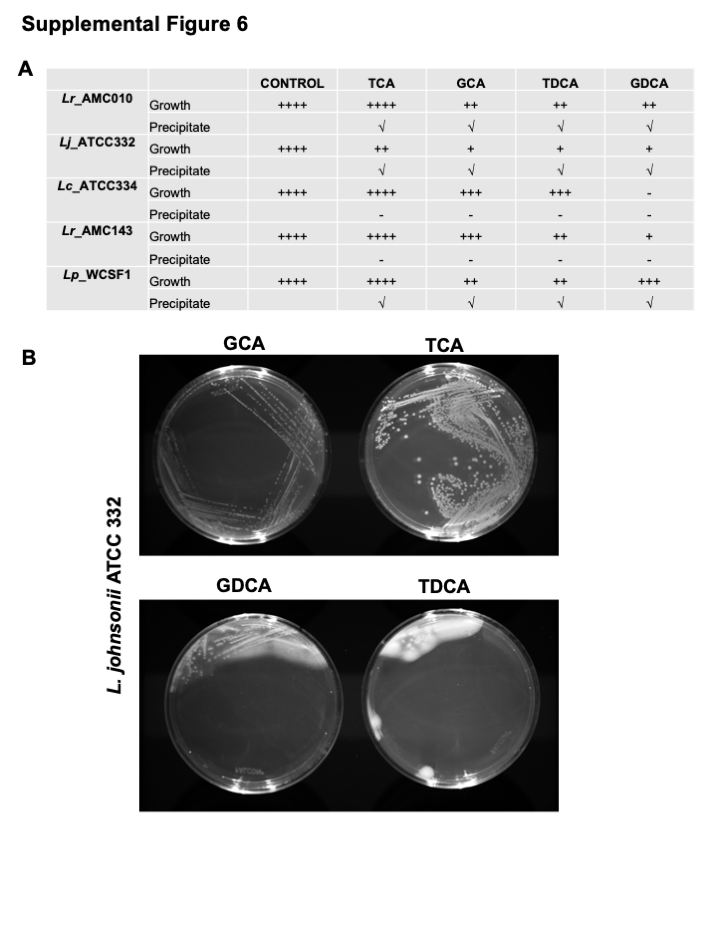

Supplement: Supplemental Material [file KGMI_A_2421623_SM1877.zip › SuppFig6.tiff]

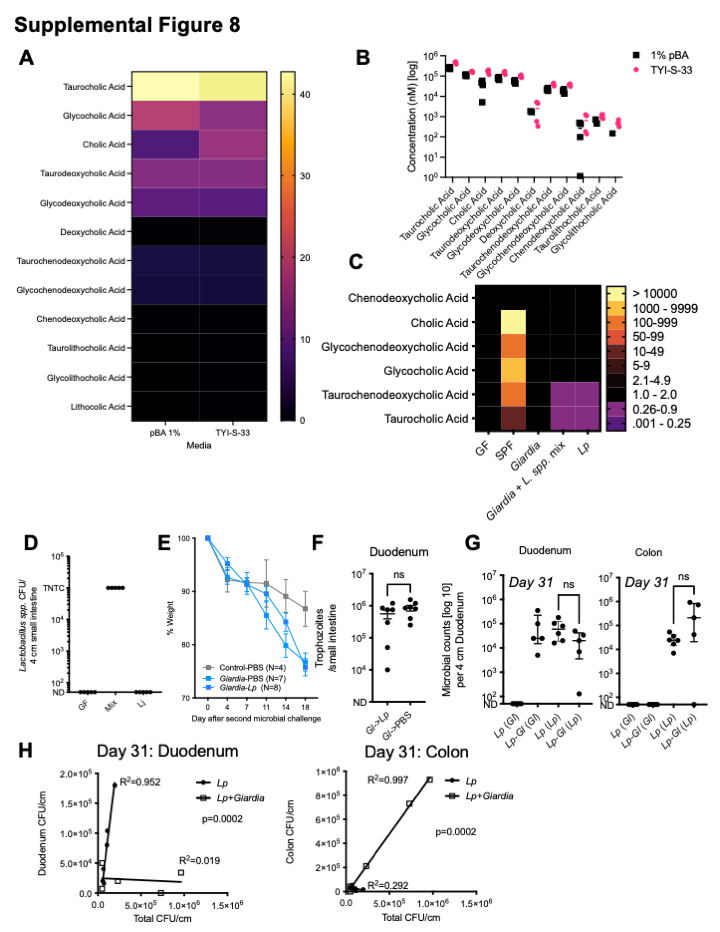

Supplement: Supplemental Material [file KGMI_A_2421623_SM1877.zip › SuppFig8.tiff]

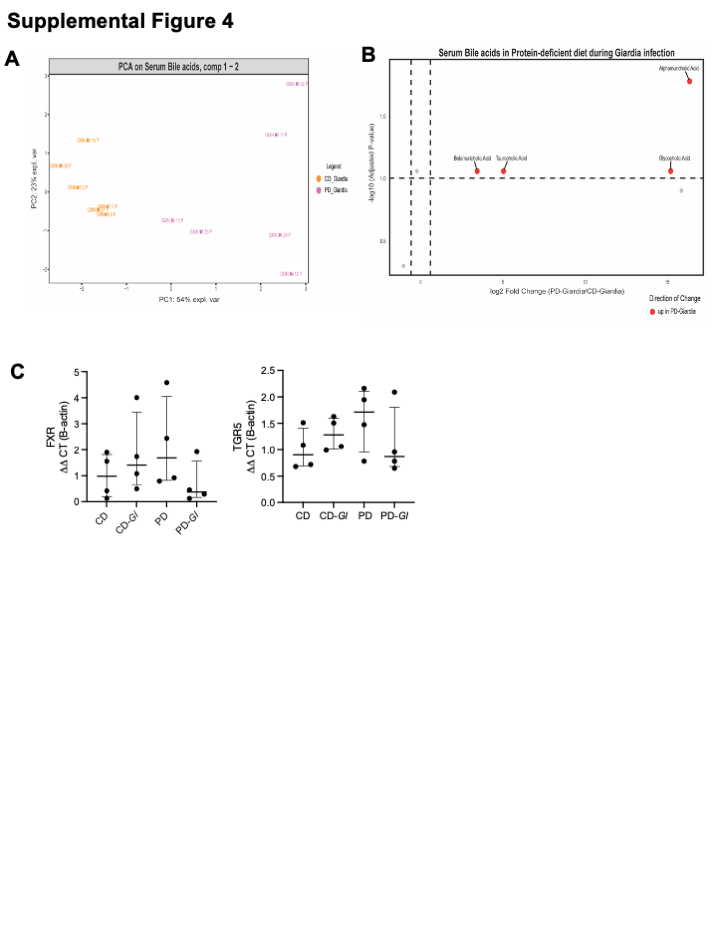

Supplement: Supplemental Material [file KGMI_A_2421623_SM1877.zip › SuppFig4.tiff]

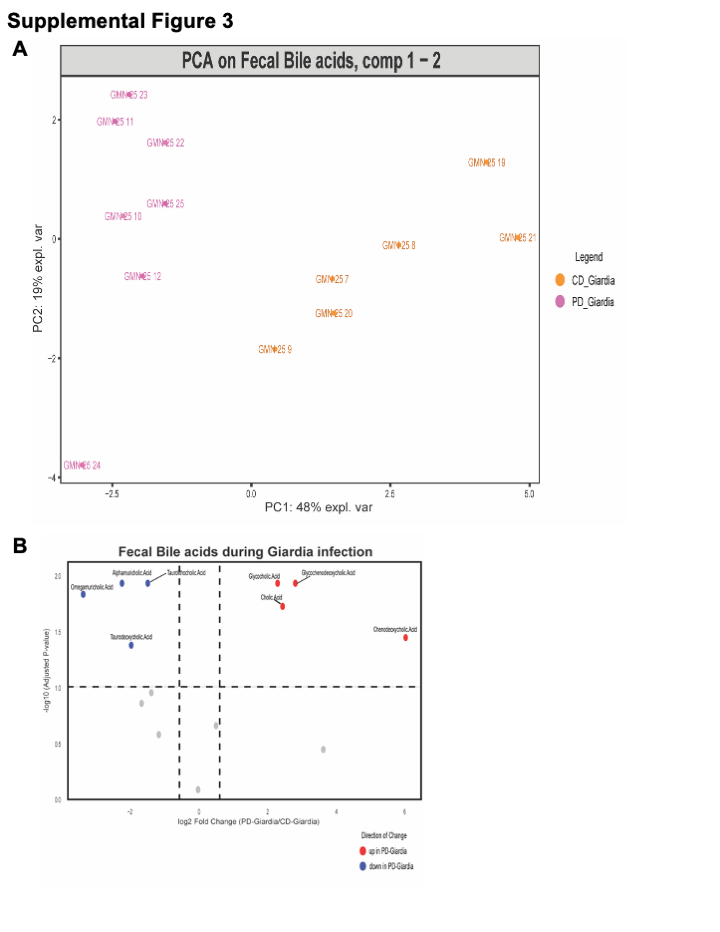

Supplement: Supplemental Material [file KGMI_A_2421623_SM1877.zip › SuppFig3.tiff]

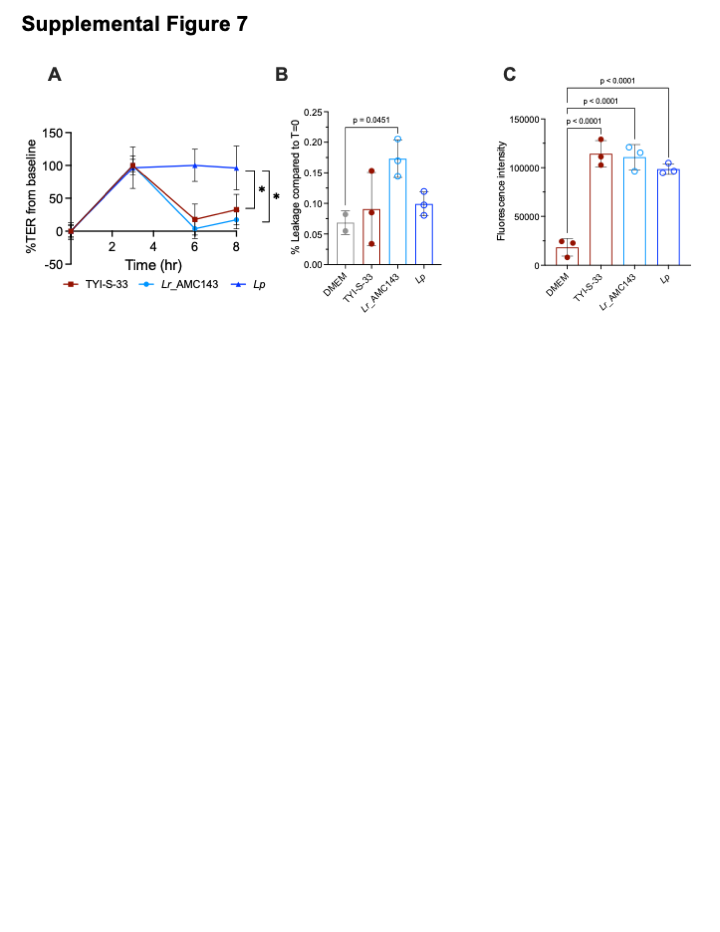

Supplement: Supplemental Material [file KGMI_A_2421623_SM1877.zip › SuppFig7.tiff]

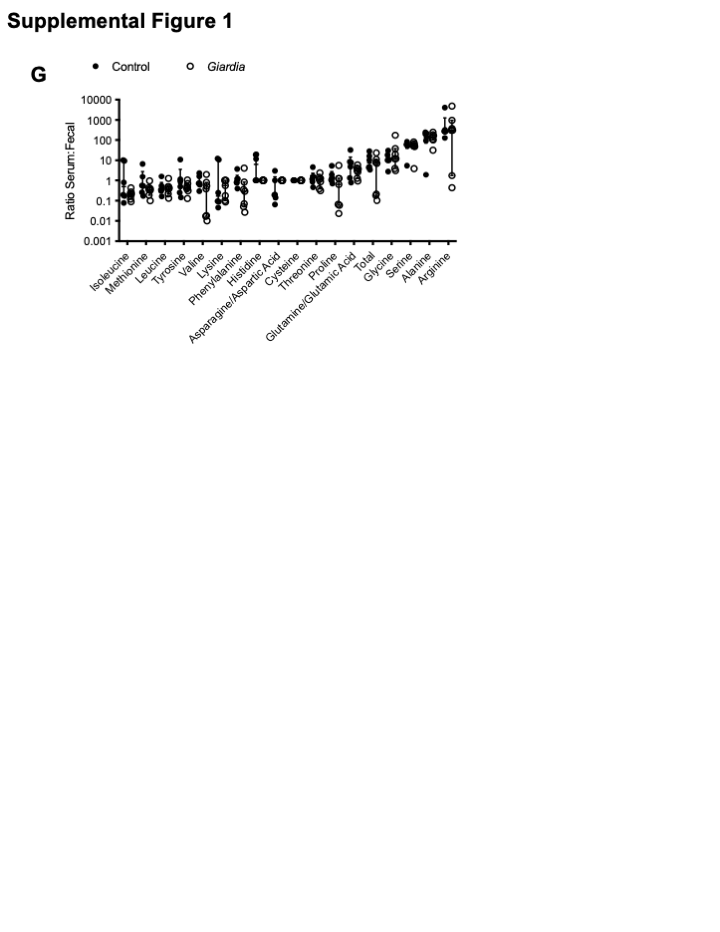

Supplement: Supplemental Material [file KGMI_A_2421623_SM1877.zip › SuppFig1g.tiff]

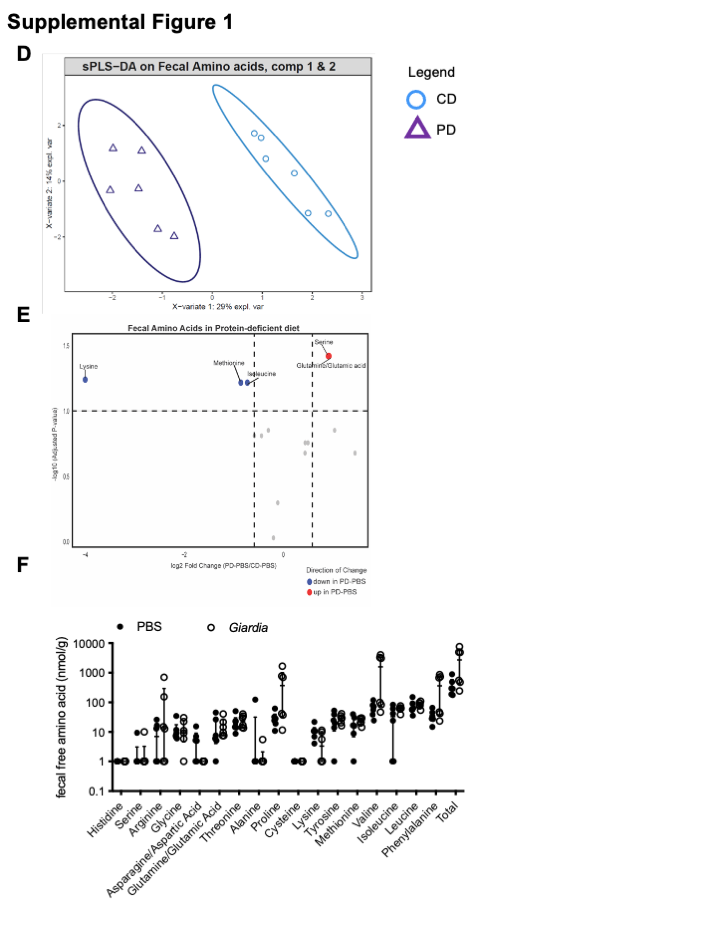

Supplement: Supplemental Material [file KGMI_A_2421623_SM1877.zip › SuppFig1d-f.tiff]
